# A High Fat “Western‐style” Diet Induces AMD‐Like Features in Wildtype Mice

**DOI:** 10.1002/mnfr.202100823

**Published:** 2022-04-28

**Authors:** Eloise Keeling, Savannah A. Lynn, Yen Min Koh, Jenny A. Scott, Aaron Kendall, Maureen Gatherer, Anton Page, Felino R. Cagampang, Andrew J. Lotery, J. Arjuna Ratnayaka

**Affiliations:** ^1^ Clinical and Experimental Sciences Faculty of Medicine University of Southampton MP806, Tremona Road Southampton SO16 6YD UK; ^2^ Biomedical Imaging Unit University of Southampton MP12, Tremona Road Southampton SO16 6YD UK; ^3^ Human Development and Health Faculty of Medicine University of Southampton Southampton General Hospital, Tremona Road Southampton SO16 6YD UK; ^4^ Eye Unit University Hospital Southampton NHS Foundation Trust Southampton SO16 6YD UK

**Keywords:** age‐related macular degeneration (AMD), blindness, high fat diet, mouse models, unhealthy foods

## Abstract

**Scope:**

The intake of a “Western‐style” diet rich in fats is linked with developing retinopathies including age‐related macular degeneration (AMD). Wildtype mice are given a high fat diet (HFD) to determine how unhealthy foods can bring about retinal degeneration.

**Methods and results:**

Following weaning, female C57BL/6 mice are maintained on standard chow (7% kcal fat, *n* = 29) or a HFD (45% kcal fat, *n* = 27) for 12 months. Animals were sacrificed following electroretinography (ERG) and their eyes analyzed by histology, confocal immunofluorescence, and transmission electron microscopy. HFD mice become obese, but showed normal retinal function compared to chow‐fed controls. However, diminished β3tubulin labeling of retinal cross‐sections indicated fewer/damaged neuronal processes in the inner plexiform layer. AMD‐linked proteins clusterin and TIMP3 accumulated in the retinal pigment epithelium (RPE) and Bruch's membrane (BrM). Neutral lipids also deposited in the outer retinae of HFD mice. Ultrastructural analysis revealed disorganized photoreceptor outer segments, collapsed/misaligned RPE microvilli, vacuoles, convoluted basolateral RPE infolds and BrM changes. Basal laminar‐like deposits were also present alongside abnormal choroidal endothelial cells.

**Conclusions:**

We show that prolonged exposure to an unhealthy “Western‐style” diet alone can recapitulate early‐intermediate AMD‐like features in wildtype mice, highlighting the importance of diet and nutrition in the etiology of sight‐loss.

## Graphical abstract

Wildtype C57BL/6 mice maintained for 12 months on a high fat “Western‐style” diet (45% kcal from fat) develop signs of retinal pathology, which are similar to patients in early‐intermediate stages of age‐related macular degeneration, the commonest form of blindness in developed societies. These findings show that an unhealthy diet alone can increase the odds of developing sight‐loss in later life.

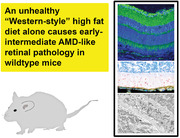

## 1 Introduction

The intake of foods with a high fat content is linked with a wide range of metabolic disorders as well as chronic illnesses including cardiovascular disease and neurodegenerative conditions such as Alzheimer's and dementia. The changing nature of human diet alongside an increasingly sedentary lifestyle has worked together to give rise to a global obesity pandemic where growing numbers of individuals experience chronic illness and associated co‐morbidities across the life‐course. Diet is also known to play an important role in the development of blinding diseases such as age‐related macular degeneration (AMD), the most common cause of irreversible sight‐loss amongst adults in developed societies.^[^ ^1^, ^2^ ^]^ Studies of human populations have shown an association between foods containing high fat dietary products, fried and refined food, and those enriched with red/processed meat, often referred to as a “Western‐style” diet, with AMD.^[^ ^3^, ^4^ ^]^ By contrast, a diet containing nutrient‐rich fresh foods such as vegetables, fruits, legumes, and fish, appear to confer some protection, in some cases by significantly reducing the incidence of AMD.^[^ ^5^, ^6^ ^]^ Consequently, based on findings of the Age‐related Eye Disease Study (AREDS) and follow‐up investigations, some AMD patients are prescribed antioxidants and zinc supplementation.^[^ ^7^ ^]^ Unraveling this complex relationship between diet and sight‐loss is therefore critical, if the growing incidence of AMD is to be addressed in an increasingly overweight and obese population that is also aging. In order to investigate the links between different types of foods and retinal degeneration, investigators have taken advantage of numerous rodent models that are either genetically modified and/or are aged. In this way, the role of specific AMD‐risk genes or combinations thereof can be studied in a background of a controlled diet incorporating the largest risk factor of age.^[^ ^8^ ^]^ Here, we used a mouse model to elucidate the role of an unhealthy “Western‐style” diet on the normal aging retina. In this study, we excluded the involvement of any specific AMD‐risk genes, to solely investigate the chronic effects of a high fat diet (HFD) by using wildtype C57BL/6 mice. This was achieved by maintaining mice for 12 months on a diet consisting of 45% kcal fat, which is considered the closest approximation to a typical “Western‐style” diet in humans.^[^ ^8^, ^9^ ^]^ Comparisons were made with littermates that were maintained in parallel for a similar period on a control chow diet typically given to rodents.

Our findings in HFD mice show the presence of several AMD‐linked proteins accumulating in the retinal pigment epithelium (RPE) and Bruch's membrane (BrM) complex. Interestingly, not all markers associated with AMD that were included in the study were upregulated. We also observed a significant build‐up of neutral lipids in chorioretinal tissues in the HFD animals. Analysis by electron microscopy revealed alterations at the ultrastructural level in the photoreceptor, RPE and BrM layers, including the appearance of focal deposits that were similar to pathogenic basal laminar deposits (BLamD) in donor human eyes, as well as abnormalities in endothelial cells lining blood vessels of the underlying choriocapillaris. However, pathology was not limited to the outer retina, as fluorescence labeling of microtubules in the inner plexiform layer (IPL) indicated possible changes to retinal neurons. Interestingly, scotopic full‐field electroretinography revealed no significant differences in retinal function between HFD versus chow‐fed mice. Collectively, our data presents an intriguing picture in which eyes of wildtype mice maintained on a HFD for 12 months displayed many AMD‐like features. These findings offer some tantalizing insights into how an unhealthy “Western‐style” diet could predispose retinopathy in an otherwise healthy aging eye.

## 2 Experimental Section

### 2.1 Animal Housing and Husbandry

All experimental procedures were approved by a local ethical review committee and conducted in accordance with personal and project licenses (PFE11A5B5) under the UK Animals (Scientific Procedures) Act (1986). Experiments also conformed to the ARVO statement for the Use of Animals in Ophthalmic and Vision Research. C57BL/6 mice were bred and maintained at the Biomedical Research Facility (BRF) at the University of Southampton, UK. Animals were maintained in conventional cages at a constant temperature of 22 ± 2 °C containing Lignocel 2/2 bedding (IPS Ltd., London, UK) and environmental enrichment on a 12/12‐h light/dark cycle, and were provided with food and water ad libitum. At 4 weeks after birth, female C57BL/6 mice were randomly assigned to be fed with either a standard chow‐based diet (RM1 diet; Special Diet Service (SDS) Ltd., UK) containing 7% kcal fat (*n* = 29) or a high fat diet (HFD) (SDS Ltd., UK) containing 45% kcal fat (*n* = 27), predominantly in the form of lard ^[^ ^10^ ^]^ (Supplementary information). All experiments were performed in the light stage of the light‐dark cycle between 9:00 and 18:00 h. Mice were euthanized at experimental end‐points via terminal anesthesia and perfusion fixation.

### 2.2 Dosage Information

A breakdown of the high fat diet was provided in supplementary information (supplementary table). This consisted of 45% kcal from fat, which was considered to most closely recapitulate the unhealthy high fat “Western‐style” human diet.^[^ ^9^ ^]^ This was provided ad libitum to mice after 4 weeks of weaning, until animals were euthanized at 12 months.

### 2.3 Full‐Field Electroretinography

For scotopic full‐field electroretinography (ERG), mice were dark‐adapted for 12 h prior to anesthesia via intraperitoneal injection with 1 mg ketamine (Bayer PLC, Reading, UK) and 0.005 mg dexmedetomidine hydrochloride (Centaur Services, Castle Carry, UK) per 10 g body weight. Upon cessation of motor function, mice were subject to ocular treatment with 2.5% w/v phenylephrine hydrochloride (Chauvin Pharmaceuticals Ltd., London, UK) followed by 1% w/v Tropicamide (Chauvin Pharmaceuticals Ltd., London, UK) for 2 min each to facilitate pupillary dilation. During anesthesia, ocular hydration was maintained via periodic application of Viscotears (Alcon, Farnborough, UK). ERG traces were recorded in two sweeps with a 2 min interval by stimulation with white LED light (6.8 cd‐s m^−2^) of 1.5 mm diameter for 1 ms, using the Generation II Image‐Guided ERG modality attachment to the Micron III Retinal Imaging System (Phoenix Research Labs, Pleasanton, CA, USA). This required mice to be connected to three electrodes; a ground electrode (tail), a reference electrode (head), and a corneal electrode (achieved via direct contact with the system's gold‐plated objective lens). Recordings were conducted inside a Faraday cage comprised of six aluminium copper mesh panels (Micro Control Instruments Ltd., Framfield, UK) to minimize external electrical interference. For consistency, *oculus dexter* measurements were conducted first, followed by *oculus sinister*. ERGs recordings were analyzed in the V3 Phoenix LabScribe ERG software suite (Phoenix Research Labs, Pleasanton, CA, USA) in which average recordings were determined from duplicate sweeps. Average A‐wave and B‐wave amplitudes along with associated implicit times (*T*_(A)_ and *T*_(B)_) were then calculated per mouse (statistical unit) for analyses. Following experimentation, animals received a subcutaneous injection with 200 µL 0.5 mg mL^−1^ atipamezole hydrochloride (Centaur Services, Castle Carry, UK) and were allowed to recover on a heat pad until righting reflexes had returned. All experiments were performed at a room temperature of 27 °C. We have provided additional details on animal welfare related to this procedure in a previous article.^[^ ^11^ ^]^

### 2.4 Perfusion Fixation of Animals

Mice were maintained up to 48–52 weeks of age when they were perfused trans‐cardially with 4% PFA in 0.9% saline. Eyes were immediately enucleated and the posterior pole removed for Hematoxylin and Eosin (H&E) staining, analysis of lipids or for confocal immunofluorescence microscopy. Sectioned chorioretinal tissues were randomly assigned to each of these groups. A separate set of eyes were prepared for electron microscopy. The experimenter was blinded to the identity of tissues for histological and electron microscopy studies.

### 2.5 Histological Analysis

Following enucleation, the posterior pole was post‐fixed in 4% PFA for 20 min and washed three times for 5 min in 1× PBS. Eyes were then cryoprotected by submerging in a sucrose gradient in PBS for 2 h at 5%, 10%, 15%, and 20% sucrose. Eyes were incubated in a 30% sucrose solution overnight and subsequently embedded in Tissue‐Tek Optimal Cutting Temperature (OCT) compound and frozen on dry ice for 20 min. Samples were stored at 20 °C prior to cryo‐sectioning. The eyes were cut in 16‐µm thick serial sections and collected consecutively on labeled slides with approximately 12 sections per slide. Mounted sections were stored long‐term at −20 °C.

### 2.6 Hematoxylin and Eosin Staining

Frozen sections were dried in an S160 incubator (Stuart, UK) at 37 °C for 1 h. The slides were soaked in Mayer's Hematoxylin for 10 min and rinsed in water for 5 min. 0.03% acid alcohol was applied for a further 10 min before being rinsed in tap water for a further 5 min. Slides were then stained in 0.5% Eosin for 5 min and rinsed briefly in ddH_2_O followed by an ethanol dehydration gradient (50%, 70%, 90%, and 100% ethanol twice). These were submerged in Xylene for 5 min and then mounted on coverslips with DPX mountant (Sigma Aldrich, UK). Slides were initially visualized at ×20 magnification on a conventional Olympus microscope with a built‐in dotSlide system (Olympus, UK), which provided an overview of the entire sample. Chorioretinal tissues were subsequently imaged at a higher (×40) magnification. Images were visualized using the OlyVIA software suite V3.3 (Olympus, UK).

### 2.7 Nile Red Staining and Analysis

A 1 mg mL^−1^ of Nile Red (Thermo Fisher Scientific, UK) stock solution was prepared in acetone. A 1:1000 working solution was made in 1× PBS and kept in the dark. Sectioned tissues were dried at 37 °C for 1 h and rehydrated three times for 5 min in 1× PBS in a light‐protected moist chamber. The slides were then incubated in the Nile Red working solution for 10 min at room temperature in the dark. These were then washed three times for 5 min in 1× PBS and incubated with DAPI (1:1000) under the same conditions as before. The slides were subsequently washed three times for 5 min in ddH_2_O and mounted on coverslips using Mowiol. Images were collected using a Leica SP8 laser scanning confocal microscope (Leica Microsystems, UK) at ×40 magnification. The green channel (excitation 514 nm) was recorded between 570 and 620 nm and showed neutral lipids, while the red channel (excitation 561 nm) was recorded between 620 and 700 nm which labels polar phospholipids.^[^ ^12^ ^]^ Z‐stacks were captured using a system‐optimized setting for each field of view. Data presented as a red/green ratio following a method described previously.^[^ ^13^ ^]^

### 2.8 Oil‐Red‐O staining and Analysis

A 1 mg mL^−1^ Oil‐Red‐O (Sigma Aldrich, UK) stock solution was prepared in 60% isopropyl alcohol. The solution was warmed to 56 °C in a water bath for 1 h. Six parts of the solution were mixed with four parts ddH_2_O and left for 10 min. This was passed through a fine filter paper to remove sediments and obtain a working solution. The slides were dried for 1 h and rinsed in 60% isopropyl alcohol for 30 s prior to staining with the Oil‐Red‐O working solution for 10 min. These were washed briefly in 60% isopropyl and rinsed in running water for 1 min. The slides were then counter‐stained with Mayer's Hematoxylin for 1 min followed by rinsing under tap water for 5 min. Tissues were mounted with DPX mountant (Sigma Aldrich, UK) and incubated at 80 °C for 20 min. The samples were imaged as described before for H&E staining. Oil‐Red‐O stained images were opened in FIJI (NIH, USA). The lipid staining, which appears as pink droplets, were counted in each of six anonymized images from each of three eyes in chow‐fed controls or HFD groups. Droplets were quantified in each image taken at ×40 magnification, where the photoreceptor layer was parallel to the edge of the image. Values were recorded in an Excel spreadsheet and imported into GraphPad Prism (GraphPad, San Diego, CA, USA). Statistical significance was analyzed using a student's *t*‐test.

### 2.9 Confocal Immunofluorescence Analysis

Frozen sections were dried at 37 °C for 1 h before being rinsed three times in 1× PBS. 1% BSA (Sigma Aldrich, UK) in 0.3% PBS‐Tween was added 1 h prior to washing and incubated overnight at 4 °C in primary antibody (**Table** **1**). Slides were kept in a light‐protected moist chamber during the incubation period after which they were washed three times in 1× PBS. They were incubated in the secondary antibody solution for 1 h and similarly washed afterwards. Samples were then incubated for 10 min in DAPI and mounted on glass coverslips using Mowiol. Slides were imaged using a Leica SP8 laser‐scanning confocal microscope (Leica Microsystems, UK) at ×40 magnification. Z‐stacks were captured using a system‐optimized setting for each field of view. Images were opened in FIJI (NIH, USA) as split channels. The green channel was converted into a 16‐bit image. Z‐stacks were converted to maximum intensity projections, which was duplicated. While one copy acted as the unaltered original, the other was used to create a binary image with the same threshold parameters used for all the images in the series. Layers in chorioretinal sections were demarcated using the brightfield channel, which was overlaid on the binary image, and the average intensity measured and recorded in an Excel spreadsheet. Data were obtained from six images from three to five different mouse eyes for each antibody label. Values were imported into GraphPad Prism (GraphPad, San Diego, CA, USA) for statistical analysis. For analysis of nuclei numbers and dimensions, the size of 10 nuclei in each image was measured. The total area of staining was calculated by thresholding the image. A region of interest was drawn around the outer nuclear layer (ONL) and the area above the threshold limit was measured, which was then divided by the average size of ONL nuclei.

**Table 1:** Primary and secondary antibodies used for confocal immunofluorescence microscopy studies

Product name	Manufacturer	Catalogue number	Clone/isotype	Species	Dilution	RRID
Anti‐β3tubulin	Abcam	ab18207	IgG, pAb	Rabbit	1:200	AB_444319
Anti‐Clusterin	Abcam	ab69644	IgG, pAb	Rabbit	1:100	AB_1267705
Anti‐TIMP3	Abcam	ab85926	IgG, pAb	Rabbit	1:200	AB_2256149
Anti‐Collagen IV	Abcam	ab6586	IgG, pAb	Rabbit	1:500	AB_305584
Anti‐ApoE	Millipore	AB947	IgG, pAb	Goat	1:200	AB_2258475
Anti‐Rabbit Alexa Fluor 594	Life Technologies	A11072	IgG, pAb	Goat	1:200	AB_142057
Anti‐Goat Alexa Fluor 488	Life Technologies	A11055	IgG, pAb	Donkey	1:200	AB_2534102

Antibodies were diluted in blocking buffer to ratios indicated in the table. Ig indicates immunoglobulin; pAb, polyclonal antibody.

### 2.10 Transmission Electron Microscopy (TEM) Analysis

Following initial fixation by perfusion, the posterior poles were transferred to a primary fixative comprising 3% glutaraldehyde, 4% formaldehyde in 0.1 M piperazine‐N,N'bis (PIPES) buffer (pH 7.2) for a minimum of 1 h. Specimens were rinsed in 0.1 M PIPES buffer, post‐fixed in 1% buffered osmium tetroxide for 1 h, rinsed in 0.1 M PIPES buffer and block stained in 2% aqueous uranyl acetate for 20 min. Samples were then dehydrated in an ethanol gradient (30%, 50%, 70%, 95%) for 10 min each, followed by absolute ethanol twice for 20 min. A link reagent acetonitrile was applied for 10 min, after which samples were incubated overnight in a 1:1 ratio of acetonitrile to Spurr resin. The following day, samples were infiltrated with fresh Spurr resin for 6 h before being embedded and polymerized in Spurr resin (Agar Scientific, UK) at 60 °C for 24 h. Resin blocks were sectioned using a Reichert Ultracut E Microtome (Leica Microsystems, UK). Semi‐thin sections were taken, stained with toluidine blue, and viewed on a light microscope to identify a region of interest. The blocks were then further trimmed and ultra‐thin sections taken, stained with Reynolds lead stain and viewed on a Hitachi H7700 Transmission Electron Microscope (Hitachi High Technology, Japan). Electron micrographs were imported into FIJI (NIH, USA) and the scales set using the scale bar acquired in TEM. For quantification of RPE and BrM thicknesses, 10 measurements were taken for each of 10 images across eight different eyes in separate animals from both dietary groups. Data were analyzed using GraphPad Prism (GraphPad, Sand Diego, CA, USA).

## 3 Results

### 3.1 Wildtype Mice Fed a High Fat Diet (HFD) over a Prolonged Period Became Obese, but Failed to Show any Marked Changes in Retinal Function

**Figure 1:**
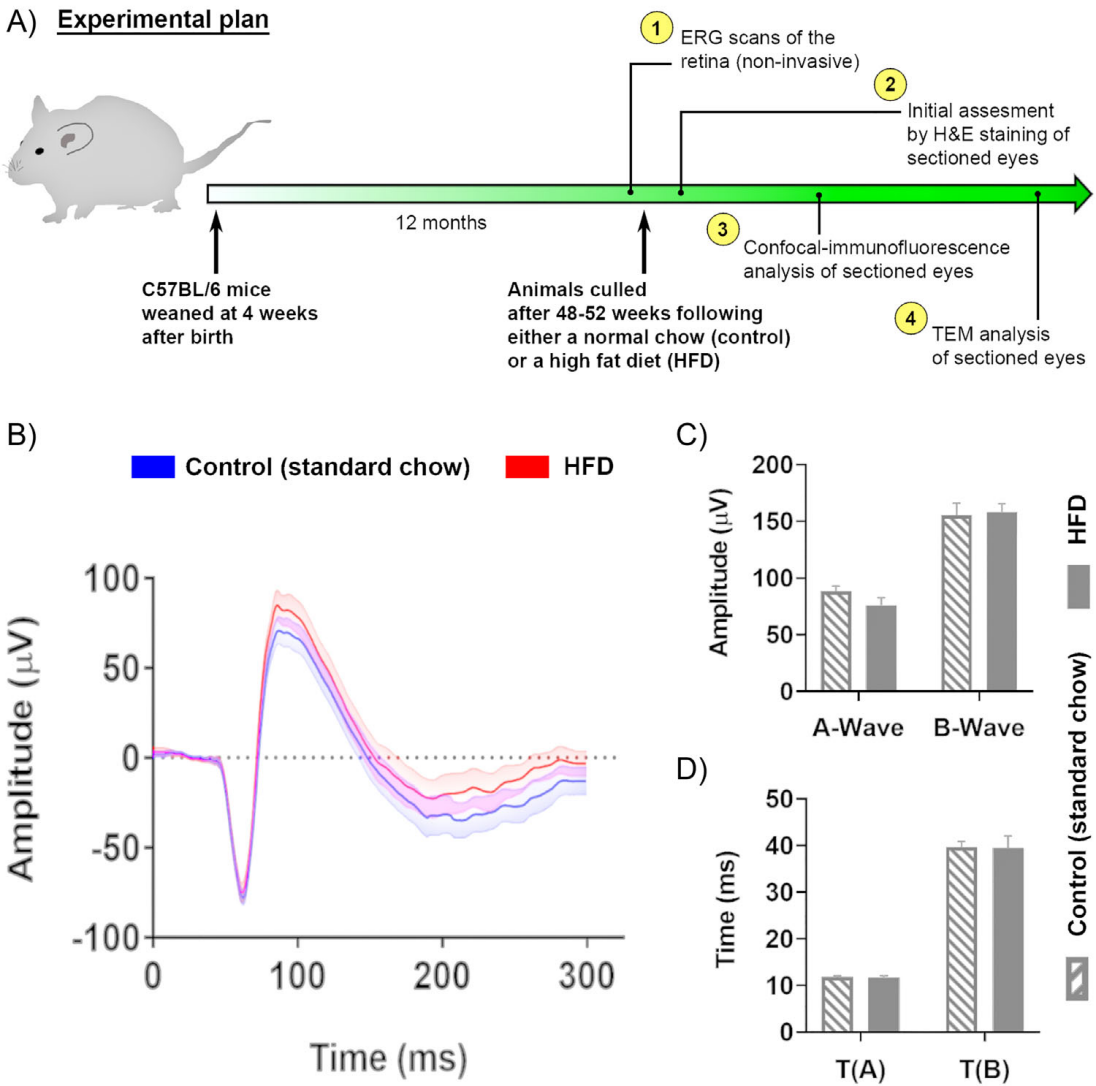
Experimental plan and assessment by electroretinography (ERG) of mice fed either a standard chow diet (control) or a high fat diet (HFD). A) Schematic diagram providing an overview of the study plan: steps 1–4. Prior to culling and collecting tissues, scotopic ERG recordings were carried out in mice from both groups. B) Averaged electrical responses are shown for mice fed either a standard diet (control: blue) or a HFD (red) with the standard deviation indicated on either side in lighter colors: *n* = 12 (chow control), *n* = 7 (HFD). C) Average A and B‐wave amplitudes, and D) average A and B‐wave implicit times (indicated as means ± SEM) showing no significant differences between groups. For A‐wave: *p* = 0.631, B‐wave: *p* = 0.132. For *T*_(A)_: *p* = 0.1569 and *T*_(B)_: *p* = 0.1516. Statistical analysis was evaluated using a student's *t*‐test.

Wildtype C57BL/6 mice maintained on a HFD for 1 year displayed marked changes to their appearance including increased body weight, an oily sheen and feel to their fur as well as alterations in circadian behavior including increased daytime activity. The C57BL/6 strain is a particularly good model for studying human metabolic dysregulation and obesity as these mice become obese and develop hyperglycemia, hyperinsulinemia as well as hypertension when fed ad libitum with a HFD. By contrast, animals remain lean and devoid of metabolic abnormalities when maintained on a standard chow diet.^[^ ^14^ ^]^ Inbred strains such as AKR/J mice exhibit resistance to insulin and are therefore less suitable for such studies.^[^ ^15^ ^]^ Other inbred mouse strains such as DBA/2 and 129T2 are also reported to have inherently different metabolic characteristics compared to the widely utilized C57BL/6 mice.^[^ ^16^ ^]^ In the current study, prior to retinal assessments and euthanasia, we recorded the total body weight in a subset of mice that were fed either standard chow or a HFD. All animals used in the study were of similar age and maintained on either one of these diets for 48–52 weeks after weaning. The mean weight of chow‐fed control animals was 33.88g ± 1.55 SEM (*n* = 12), while the mean weight of HFD fed mice was 49.23g ± 1.47 SEM (*n* = 8). The differences show a 68.8% gain of total body weight in HFD fed mice over the chow‐fed control animals within a 12‐month period (*p* < 0.0001) (Figure S1, Supporting Information). This study was undertaken over several years to minimize potential bias caused by relying on data from only a single generation of animals. Hence, different batches of mice from several litters were maintained in parallel on either diet for 12 months. Comparisons were always between HFD mice versus littermates fed the chow diet. All animals however, were subject to a common experimental procedure, which is summarized (**Figure** **1**A). Prior to culling for subsequent tissue analysis, the retinal function in a subset of mice was assessed by full‐field ERG. Non‐invasive ERG measurements can provide a robust and convenient readout of overall electrical activity in the retina, indicating functional defects in different cell types with age and/or disease. After dark adaptation, the amplitude and duration of A‐waves (derived from rod/cone photoreceptors) and B‐waves (derived from the inner retina, predominantly from ON‐bipolar and Mϋller cells) were collected following exposure of mice to a bright flash. A comparison of averaged ERG recordings was shown for the chow‐fed control (*n* = 12) and HFD mice (*n* = 7) (Figure 1B). The mean value for the A‐wave in control mice was 88.35 µV ± 4.50 SEM versus 75.92 µV ± 6.85 SEM for HFD animals (*p* = 0.631). The mean value for the B‐wave in chow‐fed control mice was 155.2 µV ± 10.8 SEM versus 158.0 µV ± 7.48 SEM in HFD animals (*p* = 0.132) (Figure 1C). The mean A‐wave implicit time for the control mice was 11.6 ms ± 0.23 SEM versus 11.60 ms ± 0.49 SEM for HFD animals (*p* = 0.1569). The mean B‐wave implicit time for the chow‐fed control mice was 39.68 ms ± 1.2 SEM versus 39.49 ms ± 2.58 SEM for HFD animals (*p* = 0.1516) (Figure 1D). The results showed no significant differences in the ERG measurements between the two dietary groups. Nonetheless, we noted a consistent decrease of A‐waves in HFD mice compared to controls, which was however outside statistical significance.

Next, the animals were culled and their eyes enucleated following perfusion fixation. The tissues were first analyzed by H&E staining to detect any obvious differences between chow‐fed controls versus HFD mice. We noticed the unusual appearance of nuclei amongst photoreceptor inner and outer segments (IS/OS) in tissues from HFD mice, which was not evident in counterpart tissues from control animals (Figure S2, Supporting Information). H&E staining also showed the patchy appearance of IS/OS in HFD eyes, whereas equivalent tissues in the eyes of the control group were labeled more uniformly. Furthermore, we observed abnormalities and potential breaks in the RPE monolayer (Figure S2, Supporting Information), which invited further scrutiny. These changes, which appeared to be limited to the outer retina, were not observed in tissues of control animals maintained on a chow diet.

### 3.2 Wildtype Mice Fed a High Fat Diet Show Changes to Inner Retinal Neurons as Well as Increased Expression of Clusterin and TIMP3 in the RPE‐BrM

**Figure 2:**
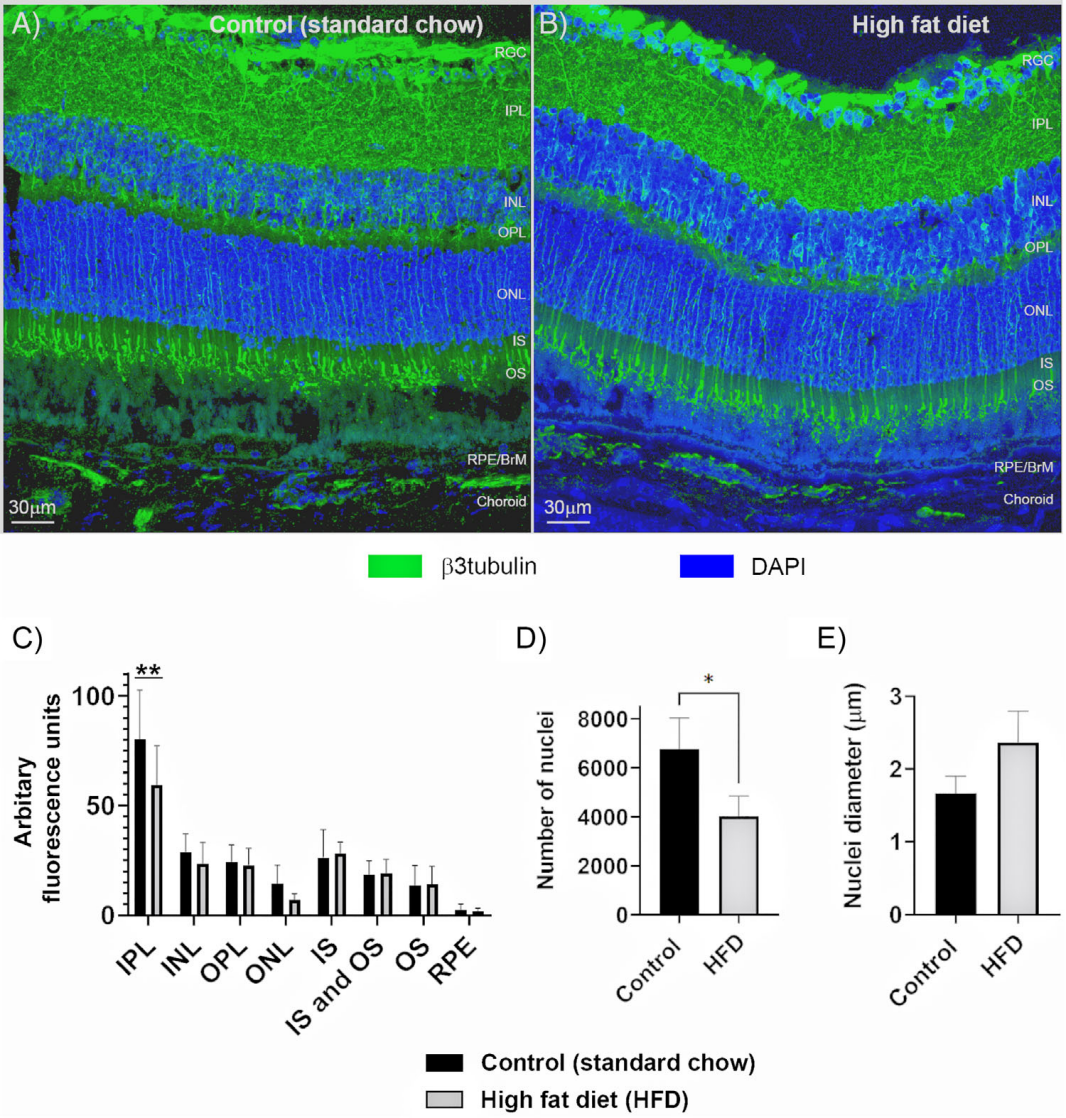
Neuroretinal organization in tissues from mice fed either a standard chow diet (control) or a high fat diet (HFD). β3tubulin staining was used to label the microtubule network of retinal neurons. A) Representative image showing a cross‐section of the retina in mice fed a control chow diet, and B) in animals fed a HFD. Scale bars correspond to 30 µm. C) Quantification of fluorescence intensity in anonymized sections from each layer of the retina show a significant reduction in β3tubulin staining in the IPL of HFD eyes (*p* = 0.0046). No differences in the extent of β3tubulin labeling were observed in the other retinal layers. Data (shown as means ± SEM) from six separate confocal z‐stack images collected from three eyes/group. Statistical analysis using Sidak's multiple comparisons test where a significance of *p* < 0.01 is denoted by **. D) Quantification of nuclei numbers in the ONL of standard chow‐fed mice versus HFD animals (*p* = 0.0348), indicting significantly fewer nuclei as a result of an unhealthy diet. E) Quantification of nuclei diameter in the ONL of standard chow and HFD mice indicated somewhat larger nuclei in the latter that were otherwise within the margin of error. Data (shown as a means ± SEM) from *n* = 3/group. Statistical analysis using an unpaired student's *t*‐test where a significance of *p* < 0.05 is denoted by *. BrM indicates Bruch's membrane; INL, inner nuclear layer; IPL, inner plexiform layer; IS/OS, inner and outer segments; ONL, outer nuclear layer; OPL, outer plexiform layer; RGC, retinal ganglion cells; RPE, retinal pigment epithelium.

**Figure 3:**
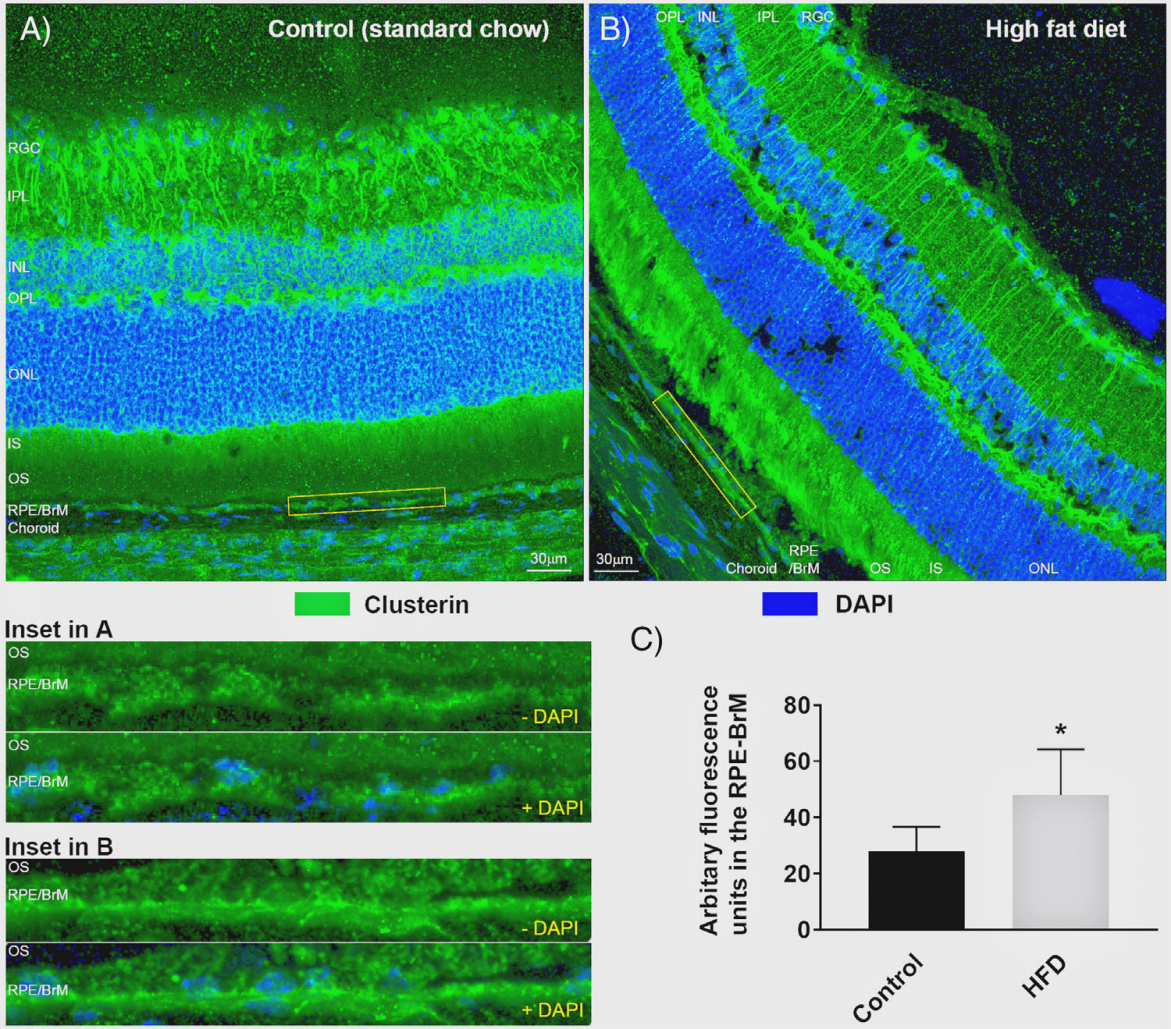
Assessment of clusterin expression in the RPE‐BrM of standard chow diet‐fed mice (control) versus high fat diet (HFD)‐fed mice. A) Representative image showing a cross‐section of the retina from mice fed a control diet and stained with clusterin, and B) from animals fed a HFD. Scale bars correspond to 30µm. Magnified images of insets (yellow boxes) from A and B showing clusterin expression in the RPE/BrM complex (without/with DAPI staining). C) Quantification of fluorescence intensity in anonymized sections in the RPE‐BrM, which showed a significant increase in clusterin staining of HFD eyes (*p* = 0.0226). Data (shown as means ± SEM) from six separate confocal z‐stack images collected from three eyes/group. Statistical analysis using an unpaired student's *t*‐test where a significance of *p* < 0.05 is denoted by *. BrM indicates Bruch's membrane; INL, inner nuclear layer; IPL, inner plexiform layer; IS/OS, inner and outer segments; ONL, outer nuclear layer; OPL, outer plexiform layer; RGC, retinal ganglion cells; RPE, retinal pigment epithelium.

**Figure 4:**
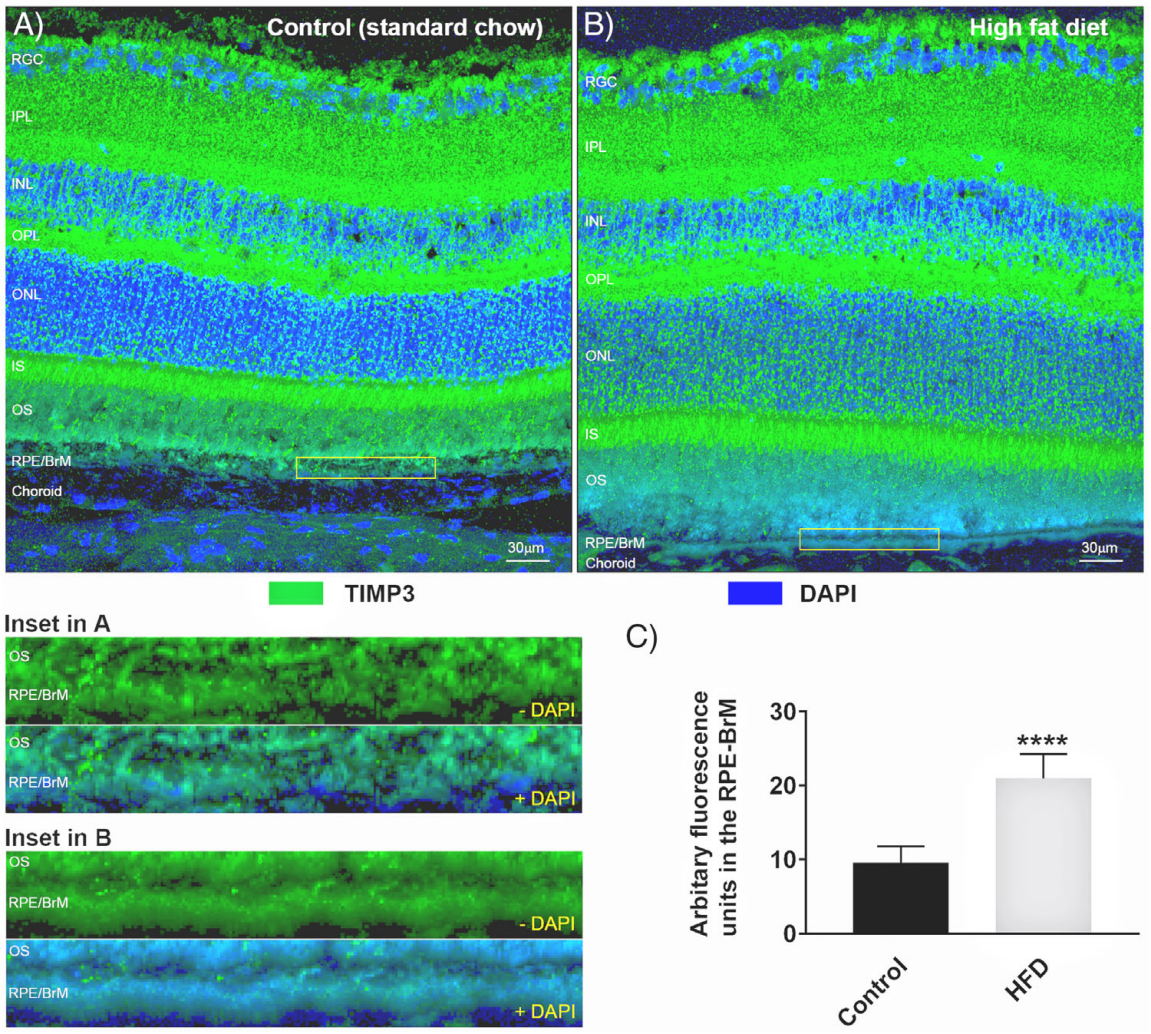
Assessment of TIMP3 expression in the RPE‐BrM of standard chow diet‐fed mice (control) versus high fat diet (HFD)‐fed mice. A) Representative image showing a cross‐section of the retina from mice fed a control diet and stained with TIMP3, and B) from animals fed a HFD. Scale bars correspond to 30µm. Magnified images of insets (yellow boxes) from A and B showing TIMP3 expression in the RPE/BrM complex (without/with DAPI staining). C) Quantification of fluorescence intensity in anonymized sections in the RPE‐BrM, which show a significant increase in TIMP3 staining of HFD eyes. Data (shown as means ± SEM) from six separate confocal z‐stack images collected from three eyes/group. Statistical analysis using an unpaired student's *t*‐test where a significance of *p* < 0.0001 is denoted by ****. BrM indicates Bruch's membrane; INL, inner nuclear layer; IPL, inner plexiform layer; IS/OS, inner and outer segments; ONL, outer nuclear layer; OPL, outer plexiform layer; RGC, retinal ganglion cells; RPE, retinal pigment epithelium.

To determine if differences between retinas of chow‐fed versus HFD mice can be discerned at higher magnification, serially cryo‐sectioned ocular tissues were stained with the neuronal marker β3tubulin/Tuj‐1 alongside the nuclear stain DAPI (**Figure** **2**A,B). Quantification of β3tubulin fluorescence in maximum intensity projections of confocal z‐stacks in anonymized tissues for each retinal layer revealed significant differences in the inner plexiform layer (IPL) between the two dietary groups. The mean arbitrary fluorescence units in the IPL of chow‐fed control mice was 80.27 ± 9.12 SEM versus 59.49 ± 7.32 SEM for HFD animals (*p* = 0.0047), indicating a significant reduction of β3tubulin labeling in the latter (Figure 2C). Quantification of fluorescence levels in the other retinal layers showed no differences between the diet groups. However, scrutiny of the DAPI stained ONL in HFD eyes showed gaps between nuclei compared to equivalent tissues in mice fed a standard chow diet. Quantification of nuclei numbers in this layer revealed an average of 6765.2 ± 732 nuclei in control eyes versus 4023.9 ± 474.7 in HFD eyes (*p* = 0.0348), indicating significantly fewer nuclei as a result of an unhealthy diet (Figure 2D). We also measured the diameter of each ONL nucleus, which had an average value of 1.67µm ± 0.13 SEM in control eyes compared to 2.36µm ± 0.25 in HFD animals (0.0698), indicating somewhat larger nuclei in the latter that were otherwise within the margin of error (Figure 2E). Next, we examined the outer retina and probed for potential changes to the expression of major AMD‐associated proteins in the RPE and the underlying BrM. The expression of clusterin was assessed in anonymized tissues from chow‐fed controls or HFD animals (**Figure** **3**A,B). Quantification of fluorescence intensity in the RPE‐BrM of control mice showed a mean value of 27.87 ± 3.67 SEM versus 48.05 ± 6.59 SEM in HFD mice (*p* = 0.0226), indicating elevated clusterin deposition in the latter group (Figure 3C). No changes in clusterin expression were detected in the other retinal layers in both control and HFD mice. We also investigated the expression of the tissue inhibitor of metalloproteinase‐3 (TIMP3) proteins in anonymized tissues (**Figure** **4**A,B). Quantification of fluorescence levels in the RPE‐BrM of chow‐fed control mice showed a mean value of 9.58 ± 0.91 SEM versus 20.98 ± 1.35 SEM in HFD animals (*p* < 0.0001), indicating a significant increase of TIMP3 expression in the latter (Figure 4C). No changes in TIMP3 expression were detected in other retinal layers of the two dietary groups. Potential changes to the major BrM structural protein collagen IV was also assessed, which yielded a mean arbitrary fluorescence value of 3.82 ± 1.09 SEM in the RPE‐BrM of chow‐fed control mice versus 2.76 ± 1.15 SEM in HFD animals (*p* = 0.51), indicating no differences between the two groups (Figure S3, Supporting Information). As expected, collagen IV expression was prominently observed around the margins of retinal blood vessels. However, suitable cross‐sections of such vessels were too infrequent in tissues to permit further scrutiny.

### 3.3 Wildtype Mice Fed a High Fat Diet Show Increased Deposition of Neutral Lipids in the Retina

**Figure 5:**
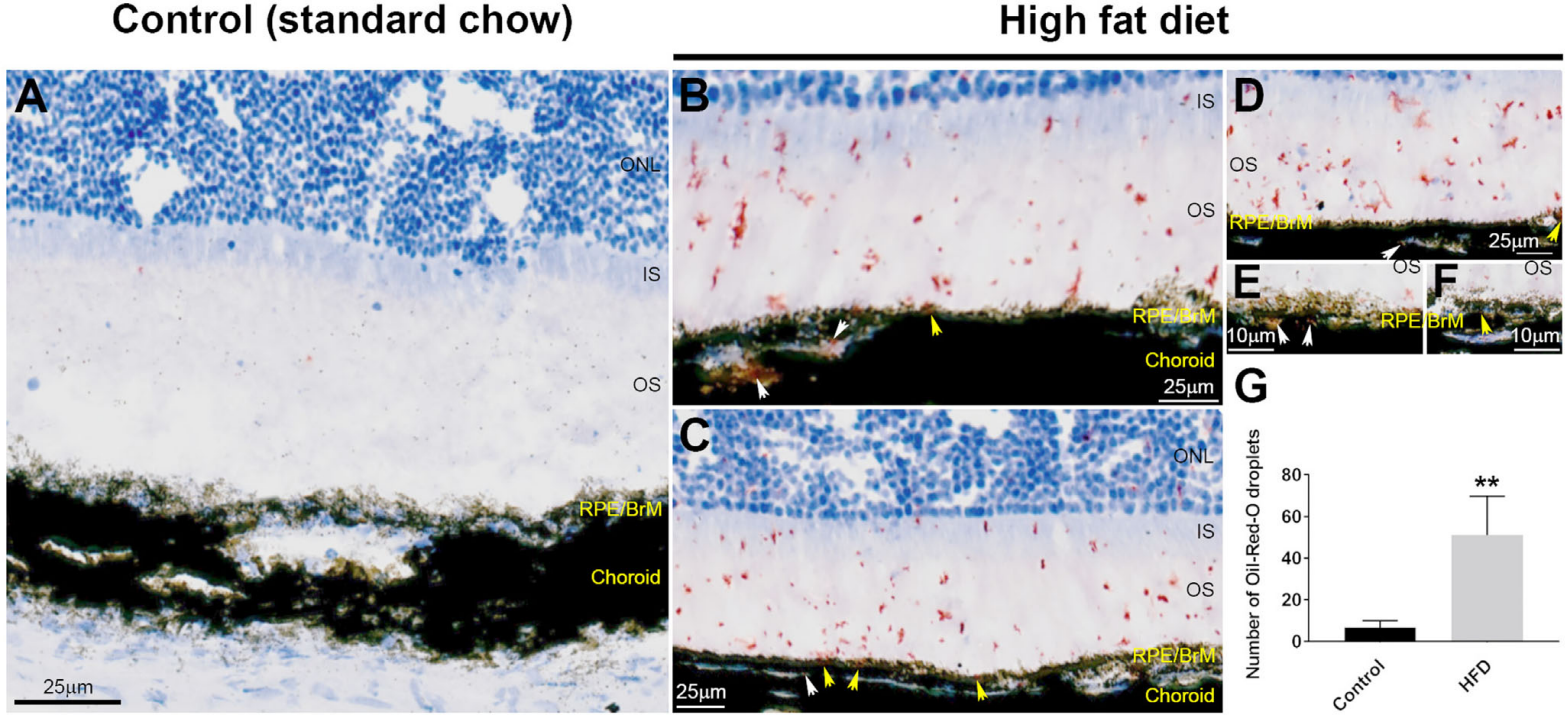
Assessment of neutral lipids in chorioretinal tissues from standard chow‐fed mice (control) versus high fat diet (HFD)‐fed mice. A) Representative image showing a cross‐section of the retina from mice fed a chow diet (control) and stained Oil‐Red‐O, and B–F) from animals fed a high fat diet (HFD). Scale bars in A–D corresponds to 25 µm and 10 µm in E–F. Although staining was predominantly evident in the IS/OS, lipid droplets could also be observed in the RPE‐BrM (yellow arrows) and in the choroid (white arrows) when the dark pigmentation in these tissues permitted their visibility. G) Quantification of fluorescence intensity in anonymized sections that show a significant increase in the number of lipid droplets (red) in HFD eyes compared to controls. Data (shown as means ± SEM) from six separate images collected from three eyes/group. Statistical analysis using an unpaired student's *t*‐test where a significance of *p* < 0.01 is denoted by **. BrM indicates Bruch's membrane; IS/OS, inner and outer segments; ONL, outer nuclear layer; RPE, retinal pigment epithelium.

As the intake of high fat and cholesterol‐enriched diets are reportedly associated with alterations to lipid metabolism, we studied the distribution of different lipids in retinas of chow‐fed control versus HFD mice. We first evaluated the expression of apolipoprotein E (ApoE) in the RPE‐BrM of chorioretinal tissues. The mean arbitrary fluorescence value in retinas of control mice was 40.87 ± 6.7 SEM versus 26.45 ± 4.0 SEM in HFD mice (*p* = 0.093), indicating no significant differences (Figure S4, Supporting Information). Nile Red (9‐diethylamino‐5H‐benzo[a]phenoxazine‐5‐one) has a high affinity for lipids and is used to detect the degree of lipid hydrophobicity via a shift in its emission spectrum from red to green. We therefore probed chorioretinal tissues with Nile Red, after which the fluorescence readouts in the respective channels were reported as a ratio. The red: green ratio in retinas of control mice was 1.20 ± 0.24 SEM versus 1.27 ± 0.13 SEM in HFD mice (*p* = 0.583), indicating no significant differences between the two groups (Figure S5, Supporting Information). Next, we evaluated the presence/distribution of neutral lipids by Oil‐Red‐O staining in retinas of mice from the two dietary groups (**Figure** **5**A–F). Quantification of lipid droplets in anonymized tissues showed a mean value of 6.67 ± 1.33 SEM for the control eyes versus 51.0 ± 7.66 SEM in HFD eyes (*p* = 0.0016), indicating a significant increase of neutral lipid staining in the latter group (Figure 5G).

### 3.4 TEM Analysis Reveals Structural Alterations to Chorioretinal Tissues in High fat Diet Mice

**Figure 6:**
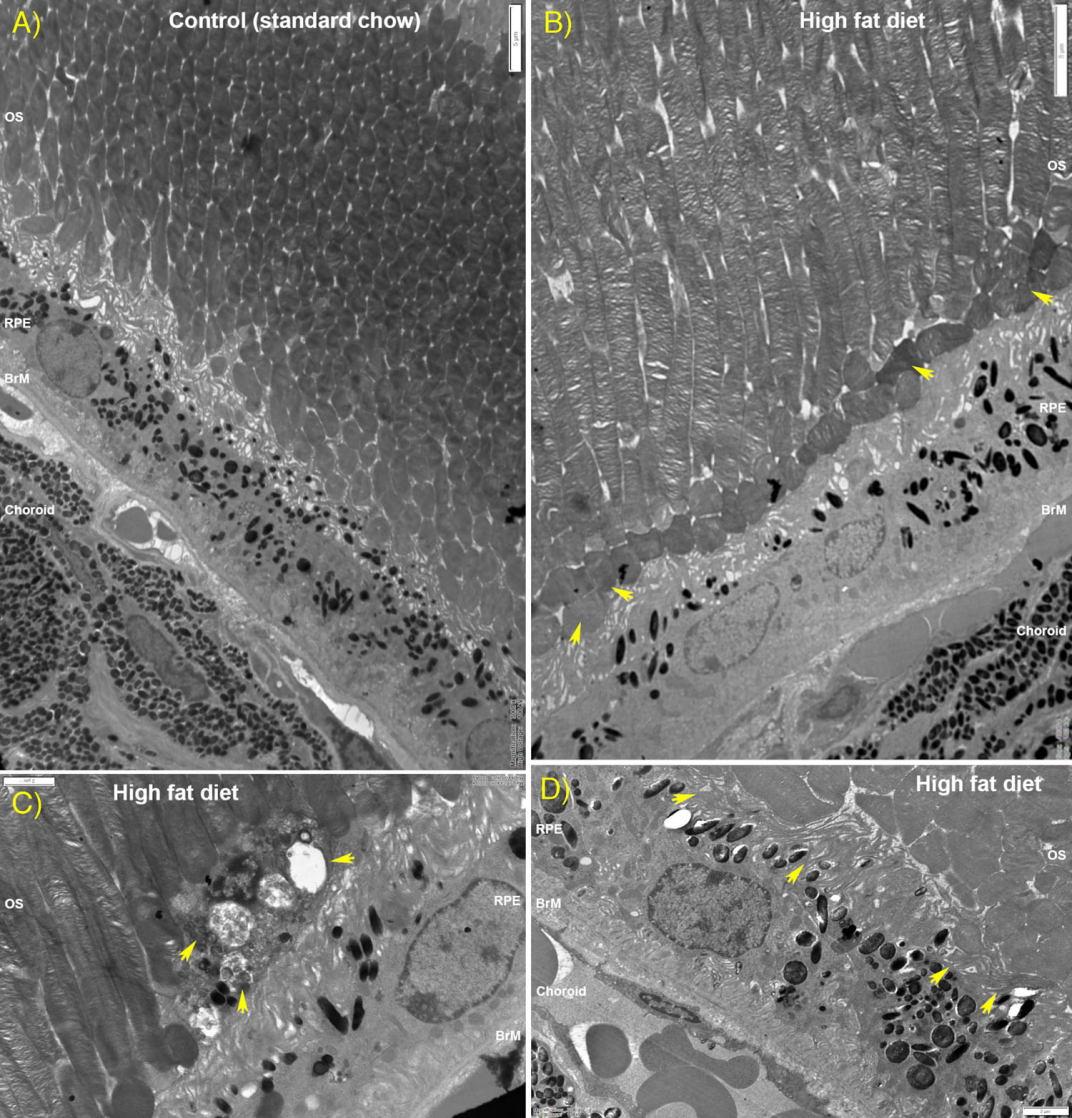
Ultrastructural analysis of photoreceptor outer segments and their interactions with the RPE in mouse eyes. A) Representative electron micrograph showing chorioretinal tissues in cross‐section from control mice fed a standard chow diet. Scale bar corresponds to 5µm. B) Counterpart tissues from eyes of high fat diet (HFD) mice, where photoreceptor OS that interact with the RPE layer are observed as disorganized structures (arrows). Scale bar corresponds to 5µm. Note the contrast with which unidirectionally angled OS in chow‐fed control mice for the most part, seamlessly interact with apical microvilli of underlying RPE cells. C) We also observed electron‐dense aggregates within vacuoles at the OS‐RPE junction (arrows) in HFD mice. These aggregates were not exclusively in vacuoles, but were also present around their proximity. Scale bar corresponds to 2µm. D) Collapsed and disorganized apical microvilli in RPE cells (arrows) were observed to correspond mainly with misaligned photoreceptor OS in HFD eyes. Scale bar corresponds to 2µm. Data from six separate images collected from three eyes/group. BrM indicates Bruch's membrane; OS, outer segments; RPE, retinal pigment epithelium.

**Figure 7:**
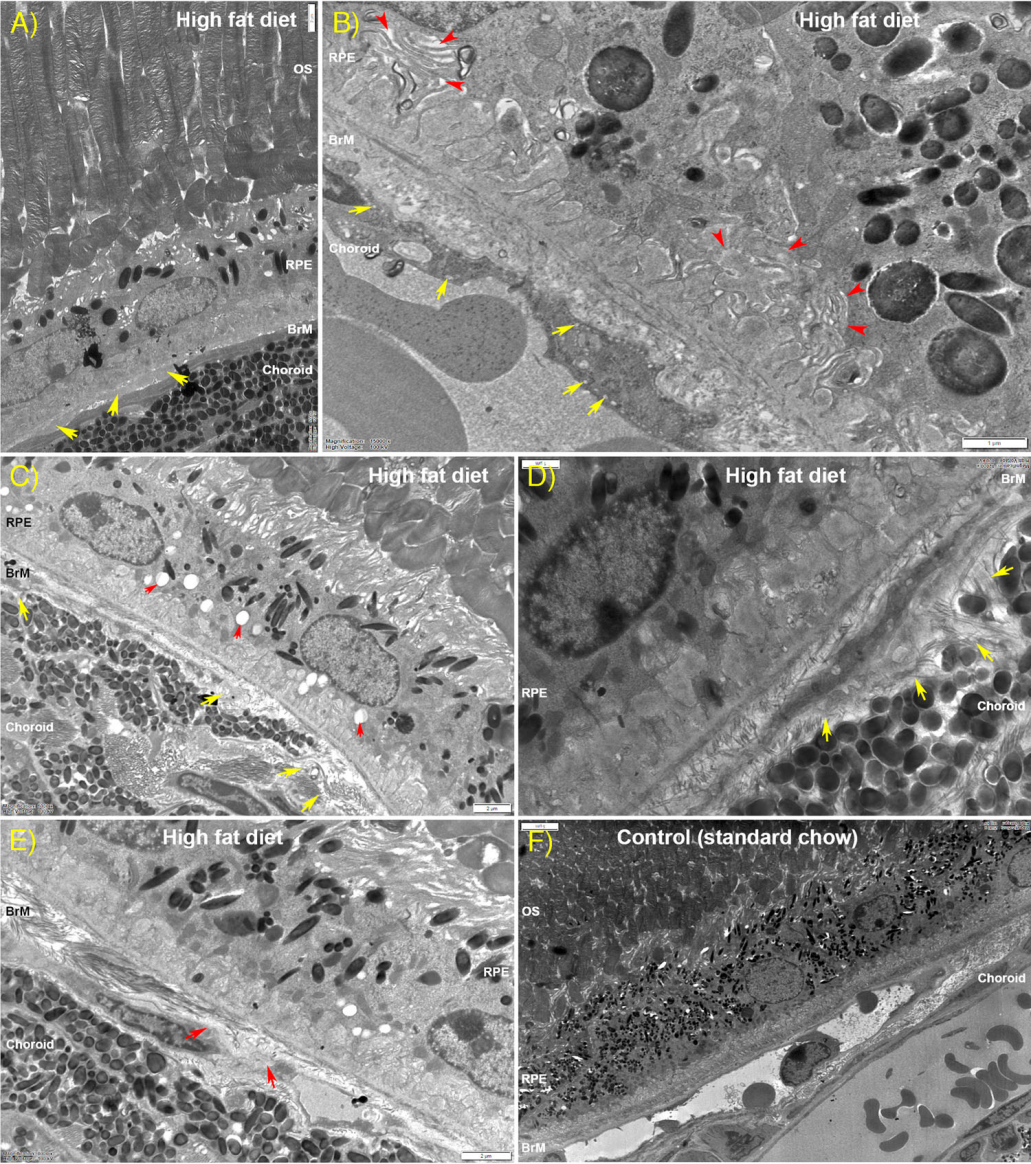
Ultrastructural analysis of RPE and associated tissues of mice fed a high fat diet (HFD). A) Transmission electron micrograph showing the presence of basal laminar (BLamD)‐like structures (arrows) under the RPE in HFD mice. Scale bar corresponds to 2µm. B) Notable invaginations of basolateral infolds in RPE cells (red arrows) could be observed in HFD tissues. On occasion, we also observed the appearance of small vacuoles within endothelial cells lining the blood vessels of the choroid (yellow arrows). Scale bar corresponds to 1µm. C) The RPE in HFD eyes contained many vacuoles compared to counterpart tissues in chow‐fed control animals (red arrows). These were predominantly observed in the mid‐lower regions of RPE cells. C–E) Indicators of structural irregularities in HFD eyes were also noticeable in the BrM, which showed some evidence of discontinuity and disorganization (arrows). Scale bars in C and E correspond to 2µm while scale bar in D corresponds to 1µm. F) A representative electron micrograph from a control mouse eye is shown for comparison. Scale bar corresponds to 5µm. Data from six separate images collected from three eyes/group. BrM indicates Bruch's membrane; OS, outer segments; RPE, retinal pigment epithelium.

**Figure 8:**
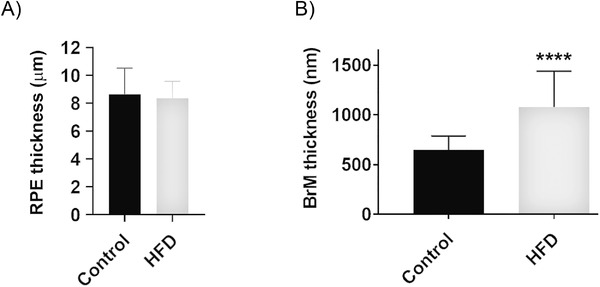
RPE and BrM thickness measurements in electron micrographs from mouse eyes. A) No differences in the thickness of RPE cells were recorded between mice fed a standard chow diet (control) versus animals fed a high fat diet (HFD). The mean ± SEM RPE thickness in chow‐fed control mice was 8.64µm ± 0.84 versus 8.36 µm ± 0.54 in HFD animals. Statistical analysis using an unpaired student's *t*‐test (*p* = 0.784). B) By contrast, a significant increase in BrM thickness was observed in HFD mice. The mean ± SEM BrM thickness measurement in chow‐fed control mice was 649.1 nm ± 13.8 versus 1077 nm ± 36 for HFD animals. Statistical analysis using an unpaired student's *t*‐test where a significance of *p* < 0.0001 is denoted by ****. Data obtained from 10 measurements from each of 10 different electron micrographs taken from eight different animals (*n* = 8/group). Samples were anonymized prior to quantification. BrM indicates Bruch's membrane; RPE, retinal pigment epithelium.

Given that ultrastructural changes in cells and tissues often precede retinopathy, we compared by TEM the retinae of HFD mice with control animals fed the chow diet. Micrographs were collected from serial sections that were cut parallel to the long axis of photoreceptors with the identity of tissues anonymized to eliminate any bias in subsequent analyses. A representative electron micrograph of the outer retina from a control animal fed the chow diet is shown, denoting its major structures (**Figure** **6**A). By comparison, the outer retinae of HFD mice showed an unusually high number of disorganized or collapsed outer segments (OS) (Figure 6B and Figure S6, Supporting Information). We also observed in these HFD mice the presence of electron‐dense aggregates organized into vesicular structures amongst OS and at the OS‐RPE interface (Figure 6C and Figure S6, Supporting Information). Some of these vesicles were contained within vacuoles while others appeared to be outside such enclosures and in the extracellular space. Interestingly, the OS surrounding such vesicles appeared to be normal. Electron‐dense aggregates were notably absent in tissues from chow‐fed controls. We also observed many instances where the apical microvilli of RPE in HFD retinas appeared to be disorganized or had partially or fully collapsed (Figure 6D). These were often but not always associated with unusually high numbers of apposed OS that were also misaligned. On occasion, we noticed the appearance of hypertrophic cells between the RPE and photoreceptor OS in HFD eyes (Figure S6, Supporting Information). Scrutiny of tissues below the neuroretina revealed the presence of focal deposits between the RPE and BrM (**Figure** **7**A). In eyes from HFD eyes, we also observed the presence of highly convoluted and invaginated basolateral RPE infolds which contained pale material between infolds, as well as the unusual appearance of small vacuoles on the luminal and abluminal faces of endothelial cells lining blood vessels of the choriocapillaris (Figure 7B). RPE cells in HFD eyes also showed a marked increase of intracellular vacuoles (Figure 7C and Figure S6, Supporting Information) as well as signs of delamination of the underlying BrM (Figure 7C–E). These features were not observed in littermates fed the chow diet (Figure 7F). Given this striking evidence of pathology, we quantified the thickness of the RPE monolayer as well as the BrM in anonymized electron micrographs. The mean RPE thickness in the chow‐fed control mice was 8.64µm ± 0.84 SEM versus 8.36µm ± 0.54 SEM in HFD group (*p* = 0.784) indicating no appreciable differences (**Figure** **8**A). By contrast, the mean BrM thickness in control animals was 649.4nm ± 13.8 SEM versus 1077nm ± 36 SEM in HFD eyes (*p* < 0.0001), indicating a significant distortion of this supportive membrane as a result of an unhealthy diet (Figure 8B).

## 4 Discussion

The link between diet and retinopathies such as AMD has become increasingly apparent, evidenced by studies carried out in large human populations, which demonstrate the harmful effects of unhealthy high fat and cholesterol‐enriched foods termed a “Western‐style” diet, as well as the protective effects conferred by “Mediterranean” or “Oriental‐style” diets.^[^ ^3^, ^4^, ^5^, ^6^, ^17^ ^]^ In order to better understand the diet‐disease axis in AMD, investigators have exploited a range of animal models consisting mainly of rodents,^[^ ^8^ ^]^ but also rabbits^[^ ^18^, ^19^ ^]^ and non‐human primates.^[^ ^20^ ^]^ Rodent models in particular offer significant advantages to studies of this kind, due to the relative ease in which human AMD‐risk genes can be incorporated, as well as the comparatively short timeframes where experiments can be performed. The loss of these benefits when using non‐human primates is offset by the inclusion of an anatomical macula, which is lacking in rodents and must be taken into consideration when interpreting findings. Several studies have examined the effects of diet in a background of specific AMD risk genes or the influences of circadian disruption, which have led to important insights.^[^ ^8^, ^21^ ^]^ Here, we investigated the effects of a high fat diet (HFD) in the retina using wildtype mice, where findings could be broadly compared with the effects of consuming unhealthy foods, irrespective of ethnicity or specific AMD risk genes in humans. Careful consideration was given to the choice of the mouse strain as well as to the type and duration of the diet. C57BL/6 mice were selected due to their suitability for studies of this kind, which includes closely paralleling the development of human metabolic dysregulation, obesity, and atherosclerosis amongst other conditions, while otherwise remaining lean and physically normal when restricted to a typical rodent chow diet.^[^ ^14^ ^]^ In the past, investigators have used a variety of modified diets to gain insights into the diet‐disease axis, which includes high fat diets consisting of 25–60% kcal from fat, some of which also includes a high cholesterol content, high fructose, calorie restriction models as well as ketogenic diets or combinations thereof.^[^ ^8^, ^22^ ^]^ The choice of selecting the most “appropriate” HFD also highlights the various tension points between a desire to closely recapitulate an unhealthy human diet versus practical considerations such as the study duration and associated costs. Maintaining rodents on increasingly higher fat content diets results in greater levels of obesity as well as shorter timespans in which this is achieved. Hence, while feeding rodents on a 60% kcal from fat diet may be desirable under some circumstances, certain caveats may outweigh apparent benefits. For instance, a study comparing the profiles of low fat (10%), high fat (45%), and very high fat (60%) diets revealed that a majority of alterations to lung metabolites occurred in high fat‐fed mice relative to control animals, with comparatively fewer changes between high fat versus very high fat diet animals.^[^ ^23^ ^]^ The typical European and American diet contains 36–40% fat by energy. Hence, a tolerable high fat human diet may contain 50–60% of energy as fat. By contrast, a 60% fat rodent diet presents a considerably greater distortion of the normal rodent diet. Hence, mouse studies using very high fat contents are not considered to be as relevant to human physiology as those using a diet consisting of 45% kcal from fat.^[^ ^9^ ^]^ Our study therefore used a 45% high fat rodent diet to closely model the effects of an unhealthy “Western‐style” human diet. Further deliberations in the design included the length of the study as well as the initial age of exposure. Studies using shorter experimental periods from days to weeks seek to recapitulate adaptive or acute responses, while longer exposures investigate chronic effects. Most investigations focus on chronic outcomes. However, a review of the literature showed that most HFD models were not maintained for as long as 52 weeks, as was the case in our study, or indeed exceed it, although studies focusing on AMD assessed longer periods.^[^ ^8^ ^]^ Another aspect of a “Western‐style” diet studied in rodents is high cholesterol. However, cholesterol is processed somewhat differently by rodents, which must be taken into consideration when interpreting findings. For instance, mice possess a higher rate of whole‐body cholesterol biosynthesis and are able to tackle dietary cholesterol challenges more efficiently compared to humans.^[^ ^24^ ^]^ Our work also utilized female mice, which a review of the literature found are used less often (only 27% in 66 studies analyzed) compared to males.^[^ ^8^ ^]^

In our study, we observed several changes to mice fed a HFD, the most notable of which was the marked increase in total body weight. A recent study that maintained C57BL/6 mice on a very high fat diet (60% kcal from fat) reported an average weight of ≈60 g after 12 months,^[^ ^25^ ^]^ which was considerably more than the average weight of HFD animals (≈49 g) in our cohort. Another study that fed C57BL/6 mice a 59.4% fat calorie diet for 12 weeks reported an average weight of ≈40 g,^[^ ^26^ ^]^ which is consistent with a faster and exaggerated rodent phenotype from diets containing a higher fat content.^[^ ^8^, ^9^ ^]^ Additional changes observed in our HFD cohort included an oily sheen and feel to their fur as well as alterations in diurnal behavior including increased daytime activity that has also been reported previously.^[^ ^27^ ^]^ Other than a consistent decrease of A‐waves in HFD mice compared to chow‐fed control animals, full‐field ERG recordings in our study revealed no significant differences in A and B‐waves or their implicit times between groups. Use of multifocal ERG in future studies however, may enable more accurate measurements that account for local areas of degeneration as demonstrated by others.^[^ ^28^ ^]^ By contrast, a previous investigation reported diminished photoreceptor‐to‐bipolar synaptic responses as well as bipolar responses in wildtype mice after only 12 weeks,^[^ ^26^ ^]^ although another study showed no differences at 12 months.^[^ ^25^ ^]^ However, both these studies used ≈60% kcal fat diets compared to the 45% fat diet in our work. Variations between exposure times (acute vs chronic treatments) may also account for these differences. Furthermore, assessment by full‐field ERGs do not always indicate evidence of pathology, even in the presence of focal retinal lesions in AMD‐like mouse models.^[^ ^11^ ^]^ This was also noted in geographic atrophy (GA) AMD patients without multifocal ERG.^[^ ^29^ ^]^

Animals were euthanized by perfusion fixation and the eyes enucleated immediately, providing the best possible source of freshly preserved tissues for subsequent analysis. We performed an initial H&E screen of serially sectioned eyes, which showed evidence of gross retinal abnormalities including patchy staining and dark pigmentation amongst IS/OS layers as well as possible breaks in the RPE‐BrM, albeit under low‐powered light microscopy. To gain further insights into potential changes caused by a HFD, chorioretinal tissues were scrutinized by confocal immunofluorescence microscopy. β3tubulin/Tuj‐1, which specifically labels neurons,^[^ ^30^ ^]^ was used to evaluate the neuroretina and any alterations to the architecture and density of retinal neurons. A class III member of the βtubulin family, the protein forms one of the two structural components of the microtubule network and has been used to assess the early differentiation of retinal neurons,^[^ ^31^ ^]^ as well as to identify types of proliferating cells in the adult retina.^[^ ^32^ ^]^ Quantification of β3tubulin labeling of retinal neurons revealed diminished levels of fluorescence in the IPL of HFD eyes compared to the eyes of chow‐fed mice. As there were no obvious morphological changes to neuronal processes in the IPL, our findings may be due to fewer or damaged axons and dendrites of bipolar and retinal ganglion cells (RGC) in HFD mice. Impaired RGCs are also a feature of diabetic retinopathy, which can be studied using diet‐induced mouse models.^[^ ^33^, ^34^ ^]^ Moreover, experimental hyperlipoproteinemia, which was induced by maintaining rhesus macaques on a diet consisting of 20% total fat with 10% cholesterol, resulted in bilateral segmental atrophy and gliosis of the optic nerves in half of the cohort.^[^ ^20^ ^]^ Recently, an unusual case of apparent nutritional optic neuropathy in a teenager was also reported in the UK.^[^ ^35^ ^]^ Collectively, these findings point towards potential damage to the inner retinal layers including the optic nerve, linked with an unhealthy diet and/or nutritional deficiency, where the HFD mouse model can be used as a tool for further investigation. Scrutiny of the ONL revealed significantly fewer photoreceptor nuclei as a result of an unhealthy diet and a propensity for somewhat larger nuclei. ONL loss is recapitulated in other mouse models of rental degeneration with photoreceptor loss also reported at variable distances distal from the edges of GA lesions.^[^ ^36^ ^]^

Given the well‐established links between the type of diet and the incidence of retinopathy, we also investigated the expression of several proteins associated with AMD. As an exhaustive scrutiny of all AMD‐associated markers was not feasible, we probed for clusterin,^[^ ^37^, ^38^ ^]^ TIMP3,^[^ ^39^, ^40^ ^]^ and collagen IV^[^ ^41^, ^42^ ^]^ as representative indicators of pathology in the mouse retina. Although alterations to AMD‐linked proteins are not always reproduced in mouse models of the disease, we detected elevated levels of clusterin and TIMP3 proteins in the retinas of HFD mice compared to chow‐fed littermates. As expected for ubiquitously expressed proteins, clusterin and TIMP3 were also observed in other retinal layers. However, differences were only evident in the RPE‐BrM complex, in line with pathogenic sub‐RPE deposits containing clusterin and TIMP3 documented in donor AMD tissues. Reliable delineation of the RPE monolayer from the underlying BrM in maximum intensity projections of chorioretinal sections was not always possible and hence these tissues are discussed together. Interestingly, the expression of clusterin and TIMP3 proteins in the RPE‐BrM of HFD eyes appeared somewhat patchy, perhaps indicative of focal deposits. Staining for collagen IV showed no differences between HFD versus control eyes, indicating no significant alteration to this protein that forms a major BrM constituent.^[^ ^43^ ^]^ Prominent collagen IV labeling was evident around tissues lining retinal blood vessels. However, the scarcity of tissues with discernible cross‐sections through retinal vessels hindered further analysis. We believe this warrants further scrutiny, as vascular pathology has been reported in other diet‐induced rodent models^[^ ^44^, ^45^ ^]^ while impairment of basement membranes lining blood vessels is a feature shared by other neurodegenerative conditions such as vascular dementia.^[^ ^40^ ^]^ Future studies will include qPCR analysis to test the upregulation of β3tubulin, clusterin, and TIMP3 at the genetic level. Studies of this kind could also act as a screen to identify targets from amongst a wider pool of other AMD risk genes for further investigation in this mouse model.

The accumulation of lipids in the retina, their subsequent modification, and associated pathology is linked with retinopathy. For instance, the build‐up of lipids in the BrM is regarded to alter its biophysical properties by increasing hydraulic resistance, which could impair the exchange of substances between the RPE and the choriocapillaris.^[^ ^43^ ^]^ Lipids including their oxidation products in the BrM also contribute to the formation of sub‐RPE deposits.^[^ ^46^, ^47^ ^]^ The daily ingestion and breakdown of OS by RPE cells is a major source of lipids. We therefore compared the lipid profiles in chorioretinal tissues of HFD versus mice fed a chow diet. Apolipoproteins play an integral role in the transport of lipids. *APOE* is associated with AMD,^[^ ^48^ ^]^ where the ApoE protein acts as a ligand for the low‐density lipoprotein receptor (LDL‐R) and is also secreted apically and basolaterally by RPE cells.^[^ ^49^ ^]^ Furthermore, ApoE is a component of sub‐retinal debris.^[^ ^50^ ^]^ We therefore probed for ApoE expression in the outer retinae of HFD mice, which showed no changes compared to mice fed a chow diet. Next, we used Nile Red, which is ideal for detecting lipids but is also sensitive to the degree of lipid hydrophobicity. The latter characteristic results in cytoplasmic membranes that largely consists of phospholipids being stained red, while lipid droplets which are composed mainly of esterified cholesterol and triglycerides are stained yellow.^[^ ^12^ ^]^ Previous studies have shown robust Nile Red staining of the human retina, BrM and choroid with the most prominent staining in the BrM. Chorioretinal donor tissues labeled with Nile Red showed predominantly yellow‐gold staining of OS with focal red/yellow puncta in the RPE‐BrM.^[^ ^51^ ^]^ We used a simple ratiometric approach described previously using Nile Red^[^ ^13^ ^]^ to compare lipid profiles of mouse chorioretinal tissues in the two dietary groups. Yellow stained OS indicated the presence of neutral lipids including non‐polar triglycerides and esterified cholesterol, with evidence of abundant focal lipid deposits in the RPE‐BrM in a background of polar phospholipids. Ratiometric data revealed no significant differences between HFD versus chow‐fed mice. We also used Oil‐Red‐O, which binds free fatty acids, triglycerides as well as esterified and unesterified cholesterols. Neutral lipids in the BrM, which increases as a consequence of aging, can be labeled with Oil‐Red‐O.^[^ ^52^ ^]^ Analysis of human donor eyes with no grossly visible drusen or pigmentary changes in the macula showed Oil‐Red‐O staining almost exclusively in the BrM.^[^ ^51^ ^]^ Oil‐Red‐O also revealed punctate staining of lipid deposits in chorioretinal tissues of mice maintained on atherogenic diets.^[^ ^53^, ^54^ ^]^ Oil‐Red‐O staining in our study revealed punctate lipid staining in IS and OS as well as in the RPE‐BrM and choroid of HFD eyes, which was in contrast to negligible/background staining in chow‐fed mice. Similar patterns of lipid staining were reported in another study, where LDL‐R^−/−^ mice fed a high cholesterol diet for 2 months also showed Oil‐Red‐O positivity in the RPE‐BrM with background staining in control animals.^[^ ^55^ ^]^ In summary, our findings showed no differences in ApoE expression or the hydrophobic character of lipids in HFD retinas compared to chow‐fed mice. It is likely that age may also influence the characteristics of lipids in chorioretinal tissues. Future studies could compare these in young versus aged mice to obtain further insights. By contrast, Oil‐Red‐O staining indicated a significant increase of neutral lipids in the outer retina of HFD mice.

To investigate any cellular and tissue changes at the ultrastructural level, sections cut from the central mouse retina of mice fed either a HFD or chow‐fed controls were analyzed by TEM. No obvious alterations could be discerned to the inner retina between the two dietary groups, confirming the absence of gross morphological changes evaluated earlier via β3tubulin labeling. In the outer retina however, we noted an unusually high number of disorganized or collapsed OS in HFD eyes compared to chow‐fed control animals. This was unlikely due to any differences in the angle of sections, which can introduce error, as tissues were cut parallel to the long axis of photoreceptors. Comparisons were also made in anonymized micrographs from multiple eyes of several mice to mitigate bias. In our previous work, serial block face scanning electron microscopy (SBF‐SEM) was used to three‐dimensionally reconstruct and study interactions between the OS and RPE from the central mouse retina. This revealed details of unidirectionally angled OS interdigitating with long apical processes of RPE microvilli in healthy eyes of young adult C57BL/6 mice (3–6 months) fed the standard chow diet.^[^ ^56^ ^]^ Our findings in the current study show that this seamless OS‐RPE interaction appears to largely persist in chow‐fed mice at 12 months, but to a lesser extent in animals maintained on a HFD for a similar duration. Disrupted OS‐RPE interactions including loss of apical RPE microvilli are indicators of retinal pathology as well as aging.^[^ ^57^, ^58^, ^59^ ^]^ Future studies using SBF‐SEM to reconstruct these tissues in 3D are likely to reveal further insights. We also observed the presence of electron‐dense aggregates organized into vesicular structures amongst OS and at the OS‐RPE interface, which may correspond to small lipid deposits in HFD eyes. However, this can only be verified using techniques such as correlative light and electron microscopy (CLEM) in future studies. Hypertrophic and dysmorphic cells were occasionally observed between the RPE and OS, which we speculate to originate from the RPE monolayer. In a study that analyzed eyes of aged rhesus macaques, RPE cells with these features were reported overlying soft drusen,^[^ ^60^ ^]^ while hyperplastic RPE is also a feature in GA eyes.^[^ ^36^ ^]^ However, such cells in HFD mouse eyes were not necessarily associated with sub‐retinal deposits. Differences between the types of sub‐retinal deposits in mice versus macaques may account for these observations. There was also no obvious evidence of RPE loss or attenuation in HFD eyes, although this was reported by others in mice given a fast‐food diet consisting 40% energy from milk fat with 0.2% cholesterol and drinking water supplemented with high fructose/glucose.^[^ ^61^ ^]^ However, we observed exaggerated basolateral RPE infolds which accumulated pale material in eyes of HFD mice. While disorganization and loss of basolateral infolds are associated with retinopathy,^[^ ^57^, ^58^ ^]^ enlargement of these structures appear to be linked with aging and unhealthy foods. For instance, enlarged/thickened basolateral RPE infolds were reported in a mouse model of accelerated aging^[^ ^62^ ^]^ and in mice fed a HFD for 30 weeks.^[^ ^53^ ^]^ RPE cells in eyes of HFD mice also contained intracellular vacuoles. Their occurrence appears to be correlated with cellular stress linked with an unhealthy diet and retinal pathology.^[^ ^54^, ^61^, ^63^ ^]^ A notable feature in HFD retinas was the presence of focal deposits between the RPE and BrM. These were observed as single foci and merged in groups, discernible as inverted dome‐shaped structures or globular foci, respectively. Their appearance and localization bear similarities with the stereotypic deposition of basement membrane material termed basal laminar deposits (BLamD), which accumulates internally to the RPE basal laminar, either replacing or incorporating the RPE basal infolds.^[^ ^64^ ^]^ The presence of BLamD is correlated with pigmentary changes and is also a prerequisite for the appearance of soft drusen that are more closely linked with progression to later stages of AMD.^[^ ^65^ ^]^ Of note, basal mounds which are also internal to the basal laminar of RPE cells and associated with BLamD in donor human retinas, were not observed in HFD mouse eyes. A recent study of human donor eyes showed that although BLamD was found in a majority of normal eyes, all eyes with early‐intermediate AMD and ≈97% of eyes with neovascular AMD contained frequent BLamD. Moreover, BLamD doubles the diffusion distance between the RPE and choriocapillaris relative to the central three layers (inner and outer collagenous layers with a central elastic layer) of the BrM alone. The study also showed that thick BLamD can be visualized in vivo by optical coherence tomography (OCT),^[^ ^64^ ^]^ suggesting the use of OCT for future studies of HFD mice. RPE cells overlying BLamD‐like structures in HFD mouse eyes appeared normal and devoid of any pigmentary changes. Indeed, RPE cells overlying BLamD in donor eyes are expected to be functional, since they also produce and sustain BLamD components.^[^ ^66^, ^67^ ^]^ BLamD in late‐onset retinal degeneration (L‐ORD) and in AMD patients show immunoreactivity for lipids, indicating these to be a lipid‐retentive matrix.^[^ ^64^ ^]^ BLamD‐like pathology is infrequently recorded in mouse models, although sub‐RPE deposits positive for L‐ORD‐linked CTRP5 and HTRA1 proteins were reported in a transgenic mouse model of this disease.^[^ ^68^ ^]^ In another study, C57BL/6 mice were fed a fast‐food diet for 9 months, resulting in BLamD, the accumulation of basement membrane‐like material with nodular extensions into basolateral RPE infolds and choroid, as well as thickened areas of the BrM.^[^ ^61^ ^]^ Even exposure to a 25% kcal fat diet for 4 months induced BLamD in mice.^[^ ^69^ ^]^ These findings together with evidence of BLamD‐like foci in HFD mouse eyes in our study demonstrates a robust link between pathogenic subretinal deposits and an unhealthy diet. The BrM abnormalities we observed in HFD mouse eyes were consistent with a variety of pathogenic features including the deposition of lipids, amorphous, and heterogeneous material as well as altered morphology such as irregular thickening and fragmentation/delamination, reported by others in wildtype or transgenic mice fed various combinations of high fat diets.^[^ ^54^, ^55^, ^63^, ^70^, ^71^ ^]^ However, the BrM also thickens as a consequence of age.^[^ ^43^ ^]^ BrM thickness measurements from the central retina of chow‐fed control mice were consistent with values that we reported in a previous study using SBF‐SEM.^[^ ^56^ ^]^ A comparison with BrM thicknesses in eyes of HFD mice showed that an unhealthy “Western‐style” diet had significantly distorted this supportive membrane. This is consistent with other reports demonstrating significant or increasing trends of BrM thickening,^[^ ^61^, ^72^ ^]^ which may be influenced by the type of HFD and/or the period of dietary exposure. Ultrastructural examination of the choriocapillaris in our HFD cohort showed the presence of vacuoles within endothelial cells lining vessels. Similar observations have also been reported in cerebral cortex vessels of a mouse model of mucopolysaccharidoses.^[^ ^73^ ^]^ Given the importance of normal perfusion to healthy retinal function and close links between choroidal abnormalities and retinal degeneration,^[^ ^36^ ^]^ the potential effects of HFD in the choroid warrant further scrutiny.

**Figure 9:**
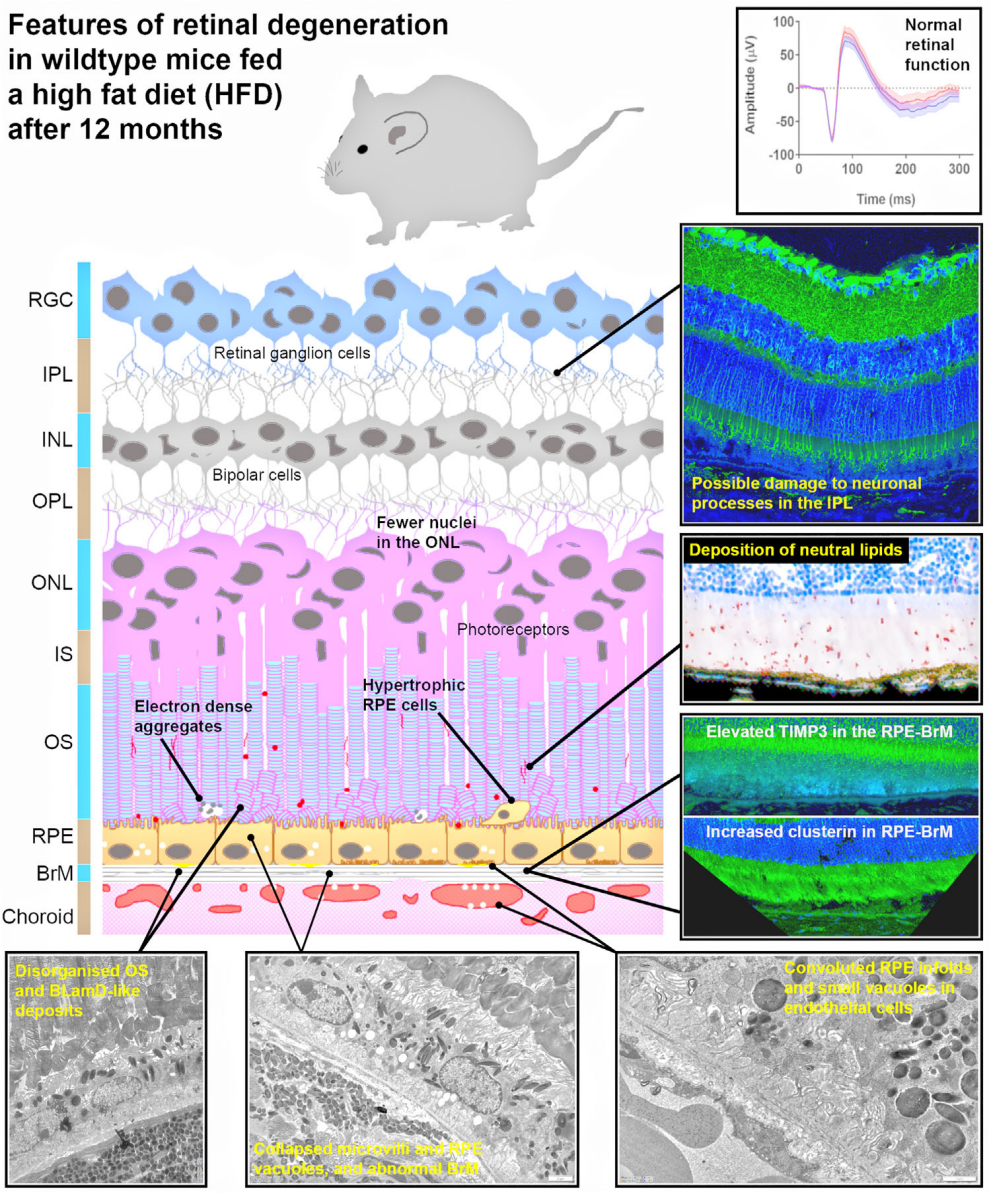
A schematic diagram summarising key findings of the study. The high fat diet (HFD) mouse model is a useful tool to elucidate the mechanisms in early‐intermediate age‐related macular degeneration (AMD), where pathogenic changes can solely be attributed to the effects of an unhealthy “Western‐style” diet.

Mouse models have been useful tools to elucidate the underlying mechanisms of retinal degeneration, which are manifold, often overlapping and/or yet incompletely defined. They allow the combination of multiple AMD risk factors of age, lifestyle (unhealthy diet and cigarette smoking etc.) with single or multiple risk genes in a single organism, within realistic timeframes and in a cost‐effective manner. Our study combined the risk factor of age with an unhealthy diet in wildtype C57BL/6 mice, so data could provide insights into understanding dietary effects without references to a specific AMD risk gene or ethnic groups influenced by effects of gene combination. A potential caveat however is the use of female only mice in experiments. Our findings show an intriguing pattern of retinal damage induced by a HFD, which is considered to most closely recapitulate the typical “Western‐style” human diet. Analysis of mouse eyes, which were preserved to the highest possible standards, and using histological, confocal immunofluorescence as well as ultrastructural imaging, showed salient AMD‐like pathology localized predominantly to the outer retina, consistent with well‐documented histopathological findings from donor eyes and retinal scans of AMD patients. Specific AMD risk proteins clusterin and TIMP3 were upregulated in the RPE‐BrM, while there was no change to the major BrM component collagen IV. Future studies could expand on these findings by profiling additional AMD‐linked proteins and genes. A HFD also directly led to the accumulation of neutral lipids in the outer retina but without any alterations to the expression of the ApoE risk protein, or wholescale changes to the degree of hydrophobicity in retinal lipids. Ultrastructural analysis revealed significant pathogenic changes throughout the outer retina, in the photoreceptor, RPE‐BrM, and the choroid akin to those reported in AMD eyes. The presence of BLamD‐like features are of particular interest as these are early indicators of retinopathy which precedes basal linear deposits (BLinD)/soft drusen associated with disease progression. These findings are summarized in a schematic diagram (**Figure** **9**). Our findings also indicate subtle changes to inner retinal neurons, though this warrants further scrutiny, particularly given the association between optic neuropathy and unhealthy/poor nutrition. Pathology in the outer retina of HFD mice shared many similarities with early stages of AMD, but without evidence of subretinal fluid accumulation, RPE loss or development of a focal lesion. However, lesion‐like pathology is reproduced in only a few rodent models.^[^ ^74^ ^]^ Hence, we propose this C57BL/6 HFD mouse model as a tool to investigate features of early‐intermediate AMD, but importantly, one which is driven by the harmful effects of an unhealthy diet alone. This is of considerable interest, as studies suggest that the odds of developing AMD due to genetic risks could be offset by dietary intervention.^[^ ^75^ ^]^ These mice therefore offer a highly tractable model to study such relationships further. Maintaining wildtype mice on a HFD beyond a 12 month period presents intriguing possibilities, as it could lead to thickening of BLamD‐like deposits and the development of ERG deficits associated with worsening pathology. Fundamental anatomical differences between humans and rodents however, are likely to preclude the natural development of a focal AMD‐lesion without additional intervention. Nonetheless, the current model provides further insights into the etiology of AMD, highlighting that diet alone is an important factor in irreversible sight‐loss.

## Author Contributions

J.A.R. conceptualized this study and supervised the research. E.L., S.A.L., Y.M.K., J.A.S., A.K. performed the experiments and collected the data. M.G. and A.P. provided technical assistance and training. A.P. also provided expertize in electron microscopy. F.R.C. provided the animals. J.A.R., E.K., and S.A.L. wrote the manuscript. A.P., F.R.C., and A.J.L. helped interpret findings and also revised the manuscript. J.A.R. provided project administration as well as acquisition of funding. All authors have read and agreed to the final version of this manuscript.

## Conflict of Interest

The authors declare no conflict of interest.

## Supporting information

Supporting Information

## Data Availability

The data that supports the findings of this study are available in the supplementary material of this article.
