# Supplementary material for: A High Fat “Western‐style” Diet Induces AMD‐Like Features in Wildtype Mice
Source: Mol Nutr Food Res. 2022 Apr 28;66(11):2100823. doi: 10.1002/mnfr.202100823 (PMC9287010; doi:10.1002/mnfr.202100823)
Supplement: Supplementary file 1 — Supporting Information [file MNFR-66-0-s001.pdf]

Supplementary information

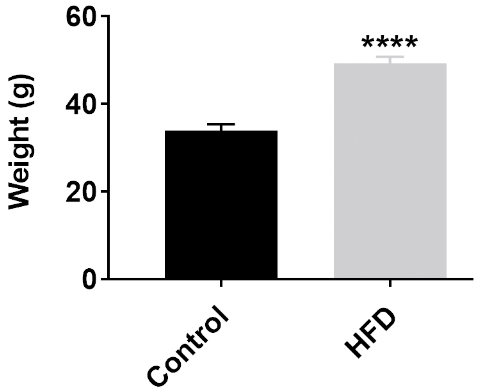

Supplementary Figure S1: Assessment of body weight in mice fed either a standard chow (control) or a high fat diet. The mean body weight of chow-fed control mice was 33.88g (n=12) compared to 49.23g (n=8) in animals fed a high fat diet (HFD). The latter group had gained significant body weight after 12 months compared to age-matched littermates on normal chow ($p<0.0001$). The data was evaluated to be normally distributed by the Shapiro-Wilk and KS normality tests, with statistical significance assessed by a two-tailed unpaired t-test where significance is indicated by ****.

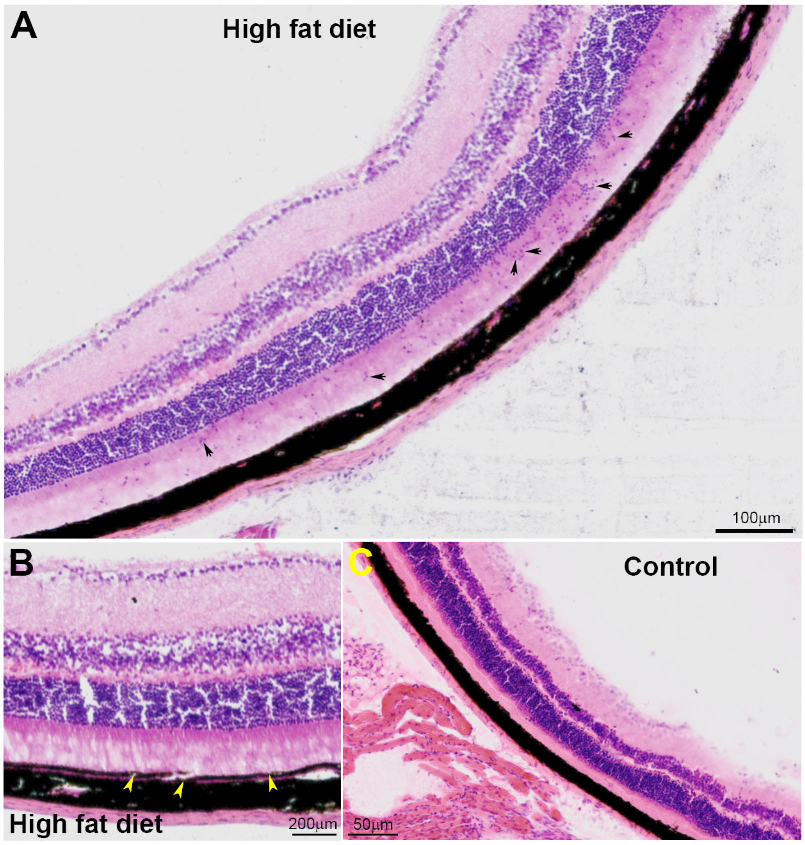

Supplementary Figure S2: Histological studies in chorioretinal tissues from mice fed either a chow control diet or a high fat diet. (A) Analysis of tissues from eyes of mice fed a high fat diet (HFD) by H&E staining showed dark pigmentation amongst photoreceptor IS and OS (arrows). (B) We also observed abnormal staining of IS and OS in eyes of HFD mice as well as potential defects in the RPE including possible breaks in the monolayer (arrowheads). (C) There was no evidence of abnormalities in retinas of littermates that were maintained on a normal chow diet. Scale bars correspond to 100µm, 200µm and 50µm, respectively in A, B and C. Data from n=10 mice/group. RGC: retinal ganglion cells, IPL: inner plexiform layer, INL: inner nuclear layer, OPL: outer plexiform layer, ONL: outer nuclear layer, IS/OS: inner and outer segments, RPE: retinal pigment epithelium, BrM: Bruch's membrane.

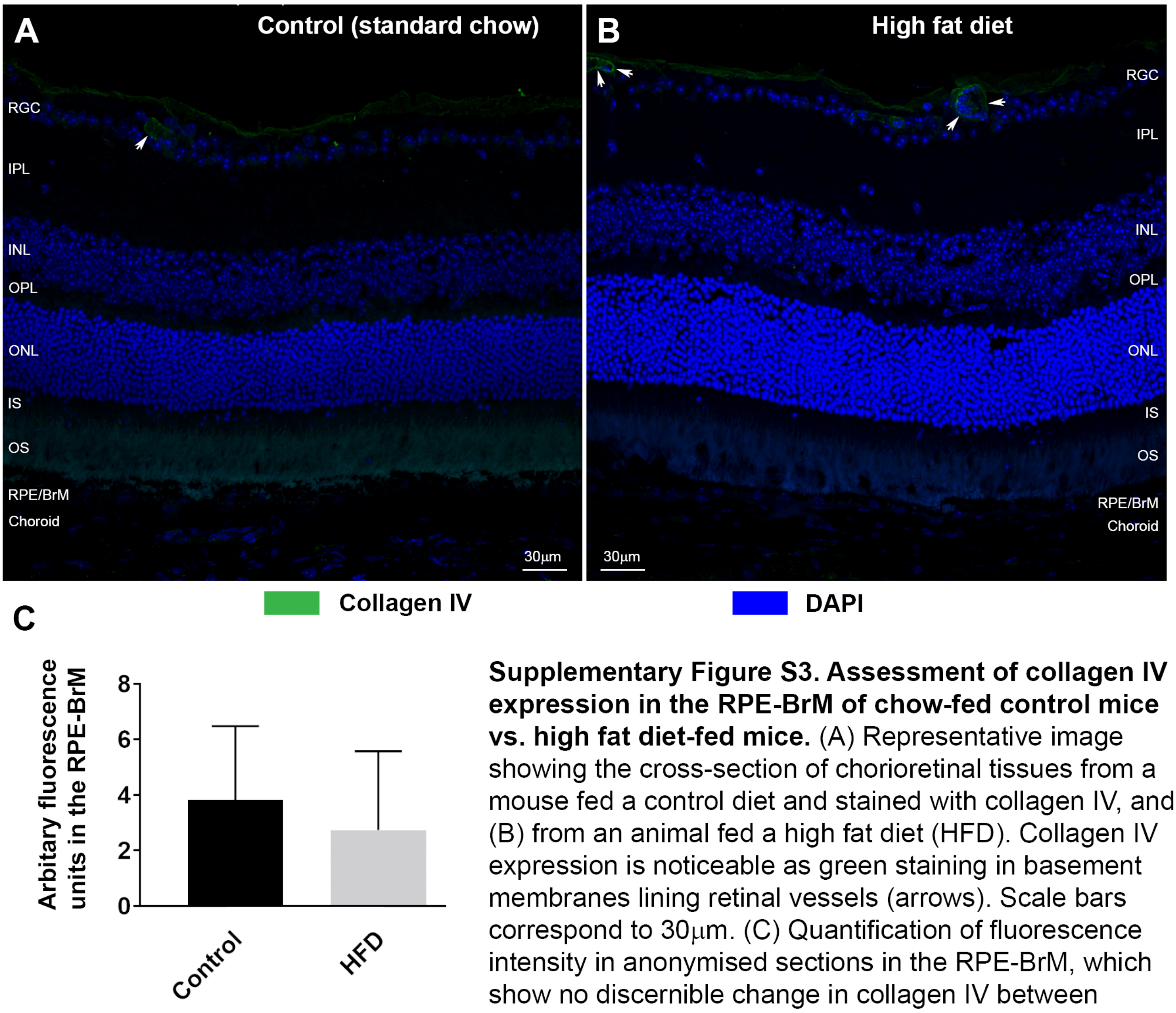

Supplementary Figure S3. Assessment of collagen IV expression in the RPE-BrM of chow-fed control mice vs. high fat diet-fed mice. (A) Representative image showing the cross-section of chorioretinal tissues from a mouse fed a control diet and stained with collagen IV, and (B) from an animal fed a high fat diet (HFD). Collagen IV expression is noticeable as green staining in basement membranes lining retinal vessels (arrows). Scale bars correspond to 30µm. (C) Quantification of fluorescence intensity in anonymised sections in the RPE-BrM, which show no discernible change in collagen IV between control and HFD eyes. Data from six separate confocal z-stack images collected from 3 eyes/group. Statistical analysis using an unpaired student's T test where $p = 0.5105$. RGC: retinal ganglion cells, IPL: inner plexiform layer, INL: inner nuclear layer, OPL: outer plexiform layer, ONL: outer nuclear layer, IS/OS: inner and outer segments, RPE: retinal pigment epithelium, BrM: Bruch's membrane.

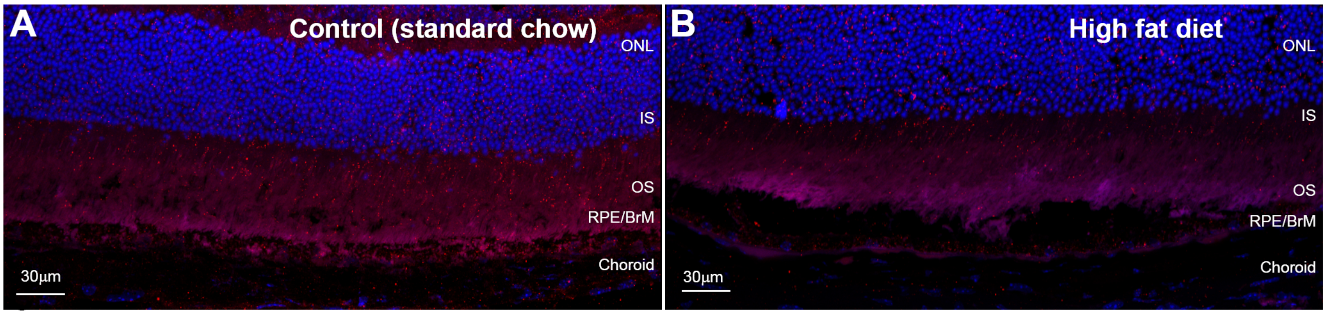

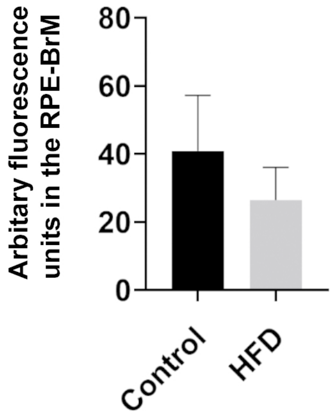

Supplementary Figure S4. Assessment of ApoE expression in the RPE-BrM of chow-fed control mice vs. high fat diet-fed mice. (A) Representative image showing the cross-section of chorioretinal tissues from a mouse fed a control diet and stained with ApoE, and (B) from an animal fed a high fat diet (HFD). Scale bars correspond to 30μm. (C) Quantification of arbitrary fluorescence units in anonymised sections in the RPE-BrM showed no measurable change in ApoE expression between control and HFD eyes. Data from six separate confocal z-stack images collected from 3 eyes/group. Statistical analysis using an unpaired student's T test where $p = 0.093$. ONL: outer nuclear layer, IS/OS: inner and outer segments, RPE: retinal pigment epithelium, BrM: Bruch's membrane.

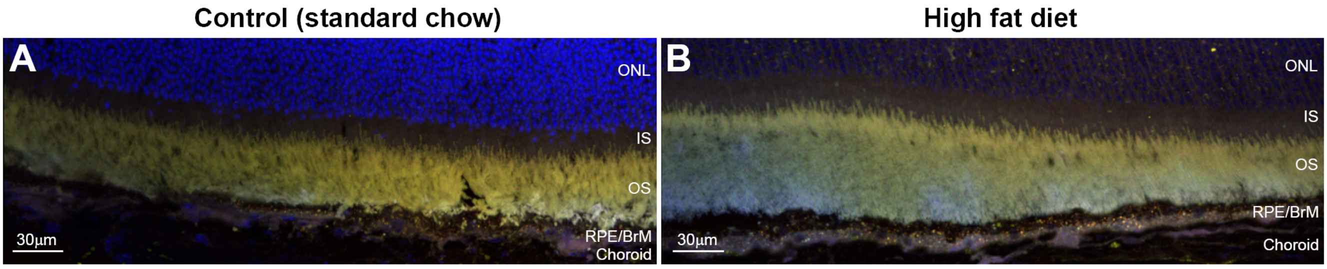

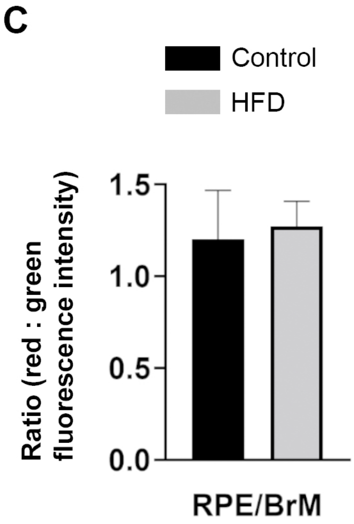

Supplementary Figure S5. Assessment of Nile Red staining of the RPE-BrM layers of chow-fed control mice vs. high fat diet-fed mice. The presence of neutral lipids and polar phospholipids was evaluated in tissues from (A) control animals vs (B) those fed a high fat diet (HFD). Data presented as a red/green emission ratio, which reports changes in the lipid composition indicating differences in hydrophobic strengths. Yellow stained OS show neutral lipids including non-polar triglycerides and esterified cholesterol, with evidence of abundant focal lipid deposits in the RPE-BrM in a background of polar phospholipids and weaker staining elsewhere. Scale bars correspond to 30μm. (C) Quantification of the red/green emission ratio in anonymised sections of the RPE-BrM indicated no measurable differences between control and HFD eyes. Data from six separate confocal z-stack images collected from 3 eyes/group. Statistical analysis using an unpaired student's T test where $p = 0.5832$. ONL: outer nuclear layer, IS/OS: inner and outer segments, RPE: retinal pigment epithelium, BrM: Bruch's membrane.

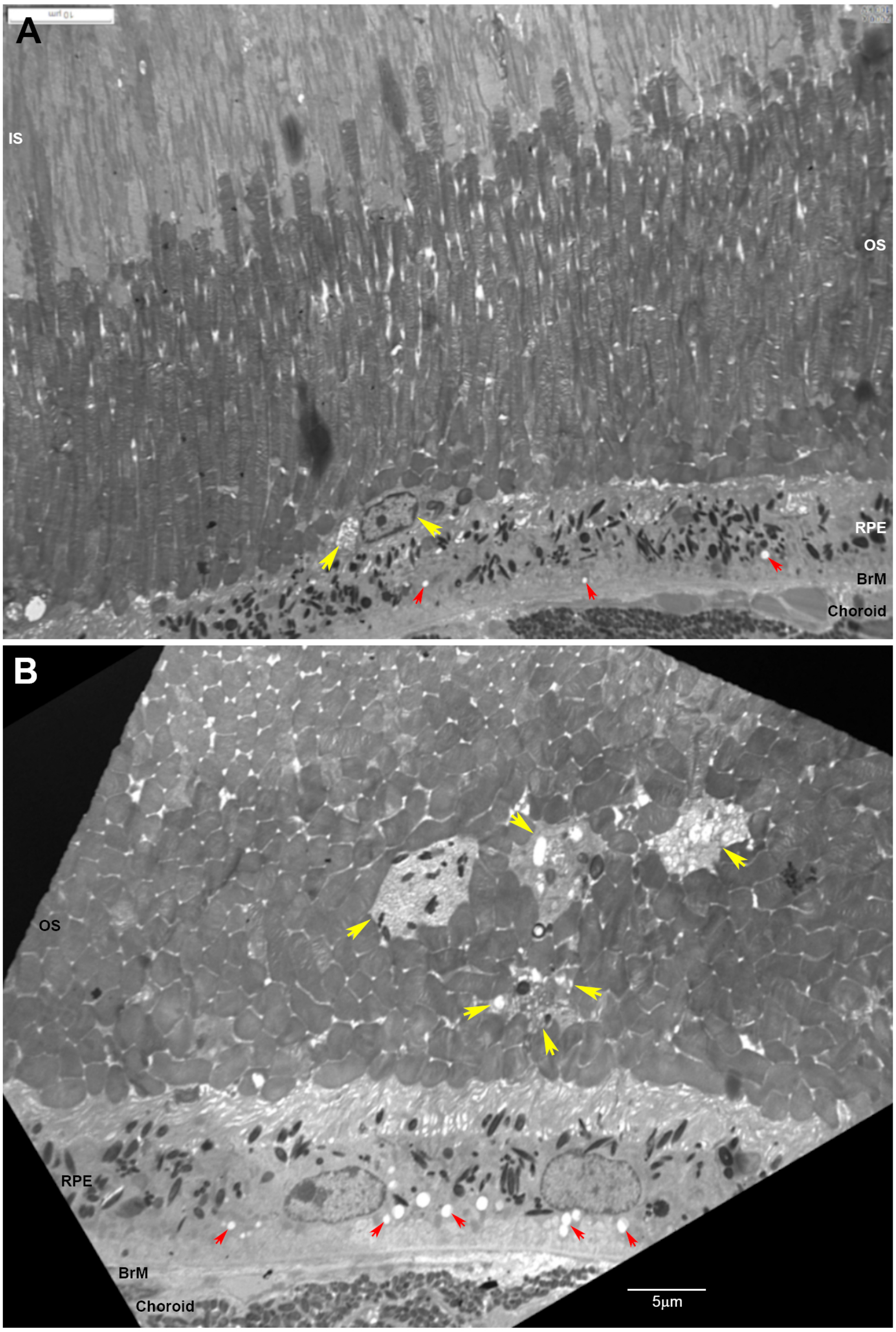

Supplementary Figure S6. Ultrastructural assessment of the outer retina of high fat diet mice. (A) Representative electron micrograph with a cross-sectional view of chorioretinal tissues from a mouse fed a high fat diet (HFD), showing further evidence of disorganised OS-RPE interactions. A hypertrophic cell (yellow arrows), presumably originating from the RPE monolayer, can be observed. Evidence of RPE vacuoles can also be observed (red arrows). Scale bar corresponds to 10μm. (B) A panned-out image of the micrograph shown in Figure 7C, showing the presence of amorphous extracellular material amongst vacuoles between OS in HFD eyes (yellow arrows). The intracellular vacuoles within RPE cells are shown for comparison (red arrows). Scale bar corresponds to 5μm. IS/OS: inner and outer segments, RPE: retinal pigment epithelium, BrM: Bruch's membrane.

Supplementary table: A comparison of nutritional composition of the diets

	**Control (% w/w)**	**HFD (% w/w)**
**Proximate Analysis**		
Crude Oil Fat	2.71%	21.22%
Crude Oil Protein	14.38%	21.55%
Crude Fibre	4.65%	4.22%
Ash	6%	4.11%
Nitrogen free extract	61.73%	37.90%
Moisture	10%	10%
**Carbohydrates, Fibre and Non starch Polysaccharides**		
Pectin	1.52%	0%
Hemicellulose	10.17%	0.11%
Cellulose	4.32%	5.56%
Lignin	1.68%	0%
Starch	44.97%	14.88%
Sugar	4.05%	21.27%
**Energy**		
Gross Energy	14.74 MJ/kg	19.05 MJ/kg
Digestible Energy	11.90MJ/kg	17.47 MJ/Kg
Metabolisable Energy	10.74MJ/kg	16.12 MJ/kg
AF Energy	3289.42kcal/kg	4287.58kcal/kg
**Fatty Acid**		
**Monounsaturated fatty acids**		
C14:1 Myristoleic	0.02%	0.02%
C16:1 Palmitoleic	0.09%	0.03%
C18:1 W9 Oleic	0.77%	6.06%
**Polyunsaturated fatty acids**		
C18:2 W6 Linolenic	0.69%	3.53%
C18:2 W3 Linolenic	0.06%	0.37%
C20:4 W6 Arichidonic	0.13%	0.01%
C22:5 W3 Clupanodonic	0.00%	0.00%
**Saturated Fatty acids**		
C12:0 Lauric	0.02%	0.03%
C14:0 Myristic	0.14%	0.29%
C16:0 Palmitic	0.31%	4.04%
C18:0 Stearic	0.04%	1.80%
**Amino Acids**		
Arginine	0.91%	0.69%
Lysine	0.66%	1.37%
Methionine	0.22%	0.53%
Cystine	0.24%	0.42%
Tryptophan	0.18%	0.19%
Histidine	0.35%	0.49%
Threonine	0.49%	0.75%
Isoleucine	0.54%	1.09%
Leucine	0.98%	1.64%
Phenylalanine	0.66%	0.9%
Valine	0.69%	1.3%

Tyrosine	0.49%	0.9%
Taurine	0.00%	0.00%
Glycine	1.11%	0.85%
Aspartic Acid	0.67%	1.22%
Glutamic Acid	3.17%	3.53%
Proline	1.20%	1.47%
Serine	0.56%	0.82%
Hydroxyproline	0.00%	0.00%
Hydroxylysine	0.00%	0.00%
Alanine	0.16%	0.69%
**Macro Minerals**		
Calcium	0.73%	0.59%
Total Phosphorus	0.52%	0.35%
Phytate Phosphorus	0.24%	0.00%
Available Phosphorus	0.28%	0.35%
Sodium	0.25%	0.15%
Chloride	0.38%	0.26%
Potassium	0.67%	0.42%
Magnesium	0.23%	0.08%
**Micro Minerals**		
Iron	159.3 mg/kg	55.70 mg/kg
Copper	11.5 mg/kg	8.22 mg/kg
Manganese	72.44 mg/kg	12.98 mg/kg
Zinc	35.75 mg/kg	64.67 mg/kg
Cobalt	634.10 mg/kg	0.00 mg/kg
Iodine	1202.69 mg/kg	238.95 mg/kg
Selenium	298.99 mg/kg	185.09 mg/kg
Fluorine	10.49 mg/kg	1.16 mg/kg
**Vitamins**		
Vitamin A	8554.27 iu/kg	4628.65 iu/kg
Vitamin D	621.70 iu/kg	1157.16 iu/kg
Vitamin E	84.10 iu/kg	94.46 iu/kg
Vitamin B1 (Thiamine)	8.58 mg/kg	5.73 mg/kg
Vitamin B2 (Riboflavin)	4.33 mg/kg	5.98 mg/kg
Vitamin B6 (Pyridoxine)	4.81 mg/kg	6.70 mg/kg
Vitamin B12 (Cyanocobalamine)	7.49 mg/kg	28.93 mg/kg
Vitamin C (Ascorbic Acid)	2.59 mg/kg	0.00 mg/kg
Vitamin K (Menadione)	10.17 mg/kg	0.89 mg/kg
Folic Acid (Vitamin B9)	0.79 mg/kg	2.22 mg/kg
Nicotinic Acid (Vitamin PP)	61.32 mg/kg	34.37 mg/kg
Panththenic Acid (Vitamin B…)	20.17 mg/kg	17.33 mg/kg
Choline (Vitamin B..	1080.14 mg/kg	1248.40 mg/kg
Inositol	2369.59 mg/kg	0.00 mg/kg
Biotin (Vitamin H)	277.13 ug/kg	231.43 ug/kg
